# Contraceptive access reform and abortion: Evidence from Delaware

**DOI:** 10.1111/1475-6773.14156

**Published:** 2023-04-09

**Authors:** Taehyun Kim, Daniel Marthey, Michel Boudreaux

**Affiliations:** ^1^ Department of Health Policy and Management University of Maryland College Park Maryland USA; ^2^ Department of Health Policy and Management Texas A&M University College Station Texas USA

**Keywords:** abortion rates, contraceptive policy, contraceptive reform, synthetic control, unintended pregnancy

## Abstract

**Objective:**

To examine the effects of a comprehensive contraceptive access reform, Delaware Contraceptive Access Now, on abortion—one of the most common outcomes of unintended pregnancy.

**Data Source:**

We used abortion data by state of residence from the Abortion Surveillance System, published by the Centers for Disease Control and Prevention. Our data covers 5 years prior to (2010–2014) and 5 years after the intervention (2015–2019).

**Study Design:**

We used synthetic control methods to estimate program effects. Our design compares Delaware to a weighted average of 45 control states (“synthetic Delaware”), where the quality of the comparison is assessed by its similarity to Delaware in pre‐period outcome levels and trends.

**Data Collection/Extraction Methods:**

Not applicable. We relied on secondary sources.

**Principal Findings:**

We did not find statistically significant evidence that the program reduced abortion rates (0.61 fewer abortions per 1000 women, *p*‐value = 0.74) on average, during the intervention period. The treatment effects were slightly larger in 2016 and 2017 (1.97 fewer abortions per 1000 women but not statistically significant) and attenuated in 2018 and 2019. This does not rule out program benefits in easing barriers to contraceptive methods or in reducing unplanned births. However, findings do suggest that increasing contraceptive access might not be an adequate substitute for restricted abortion access resulting from Dobbs v. Jackson Women's Health Organization.

**Conclusions:**

Our results suggest that comprehensive efforts to improve contraceptive access may not reduce the need for accessible and affordable abortion care.

**What is known on this topic**

- Several US states, including Delaware, have implemented comprehensive contraceptive access reforms intended to improve access to all contraceptive methods and reduce the rate of unintended pregnancy.
- Previous studies found that those reforms increased the use of effective methods.
- Research on the causal effects of a state‐based comprehensive contraceptive access reform on abortion rates is limited.

**What this study adds**

- This study estimated the causal effects of a statewide contraceptive access reform in Delaware.
- The contraceptive program in Delaware did not have a statistically significant effect on the abortion rate of Delaware residents.

## 1 INTRODUCTION

Almost half of pregnancies are unintended in the United States.^1^ Unintended pregnancy rates in the US were estimated as 32–38 per 1000 women aged 15 to 49 years in 2015 to 2019.^1^, ^2^ Unintended pregnancies that result in births impose especially high burdens. They are associated with maternal and infant morbidity and adverse economic outcomes for pregnant persons and their families.^3^, ^4^

A key strategy for reducing the risk of unintended pregnancy is providing affordable and accessible contraceptives.^5^ A handful of states have taken comprehensive approaches to promote access to all contraceptives, and long‐acting reversible contraceptives (LARCs) in particular, by implementing coordinated programs that combine public payment reform, provider training, and public awareness campaigns.^6^ These coordinated programs build on evidence from a cluster‐randomized trial that showed that clinical training and financial subsidies are effective at increasing LARC initiation and reducing unintended pregnancy.^7^, ^8^ They have also been influenced by the Contraceptive CHOICE Project in St. Louis. The program, which took place from 2007 to 2011, provided standardized contraceptive counseling to participants and reversible contraceptive methods of participants' choice at no cost.^9^ Analyses of CHOICE suggest that the program was associated with increased LARC uptake and reduced rates of unintended pregnancy.^9^, ^10^, ^11^, ^12^

The most well‐known of the statewide coordinated programs, the Colorado Family Planning Initiative, was associated with increased use of effective contraceptives, decreased fertility rates among younger persons, and improved educational outcomes.^13^, ^14^, ^15^, ^16^ Kelly et al. (2020) found suggestive yet inconclusive effects on abortion rates. An evaluation of another comprehensive program in Iowa suggests that increased LARC use after the initiative was associated with declines in abortion rates.^17^

This study evaluated the effects of Delaware Contraceptive Access Now (DelCAN), a comprehensive program in Delaware, on abortion rates. The state has had relatively high rates of unintended pregnancy, 48% in 2014.^1^ In Delaware, 39% of unintended pregnancies resulted in abortions in 2014.^1^, ^18^ As such, the effect of DelCAN on abortion incidence is an important marker of program success.

### 1.1 Program design and existing evidence

The DelCAN program sought to provide same‐day access to all contraceptive methods for free or at low‐cost to all reproductive‐aged people who can get pregnant in Delaware.^19^ The public‐private initiative was implemented during 2015 to 2020. In 2015, Medicaid payment for postpartum LARC was carved out of the global labor and delivery fee, and the Title X system received increased funding to purchase LARCs. The Title X program provides reproductive and preventative health services for low‐income populations and is the only federal program dedicated to family planning in the United States.^20^, ^21^ LARC device fees were also carved out of the Federally Qualified Health Centers (FQHC) prospective visit rate in 2017, and centers were provided devices from the state pharmacy to build their stock as they transitioned to the new payment system. Training in person‐centered contraceptive counseling (including querying patients on pregnancy desires), LARC device insertion/removal procedures, and referral for contraceptive services was conducted by the private implementation partner from 2016 to early 2019. Training included clinicians, support staff, and community organizations. Technical assistance to clinic sites for billing and coding, electronic medical records, and performance improvement continued through early 2020. A public awareness campaign, which focused on increasing attendance at reproductive health clinics, ran from 2017 to 2018. More detailed information on the program is provided elsewhere.^19^

The public awareness campaign was found to have increased Title X clinic attendance among women ages 18 to 29.^22^ A separate study suggested that LARC use increased among Title X patients by 3.2 percentage points (a 40% increase from baseline).^23^ Increased LARC initiation has also been observed in Medicaid claims data^24^ and in claims from large employers.^25^ Analysis of the Pregnancy Risk Assessment Monitoring System (PRAMS) suggests that LARC use increased among postpartum patients.^26^ However, results for other method types have been mixed. For example, existing work has not found statistically significant evidence of changes to other method types in Title X.^23^ In Medicaid, moderate method use increased for 15 to 18 year olds, but decreased for older patients.^24^ Among large employers, estimates of changes to other method types were inconclusive.^25^

Changes in contraceptive use patterns associated with the program suggest that DelCAN may have reduced unintended pregnancies, including those that would have resulted in abortion. The private implementation partner, Upstream USA, published a report showing that the abortion rate in Delaware declined by 37% between 2014 and 2017.^27^ This report has been cited in the popular press as suggesting that increasing contraceptive access is an effective strategy for reducing abortion rates, a possibility that has important implications in the context of abortion bans that will result from *Dobbs v Jackson Women's Health Organization*.^28^

While Upstream's report is suggestive of a DelCAN effect on abortions, their report was based on abortions occurring in Delaware, not abortions among Delaware residents. Abortion access in Delaware is relatively strong, and it is a small state surrounded by large non‐Delaware population centers. As a result, the occurrence abortion rate in Delaware is strongly influenced by changes in the number of out‐of‐state patients who might travel to Delaware and the number of in‐state patients who might travel outside Delaware as policies in neighboring states change. For example, based on the CDC Abortion Surveillance data,16.0% of abortions occurring in Delaware were to out‐of‐state patients in 2014, which decreased to 11.8% in 2017. In addition, the proportion of Delaware residents' abortions occurring in other states has increased over time, from 15.5% in 2014 to 31.4% in 2017.^29^ The Guttmacher Institute estimates an even higher out‐of‐state utilization rate among Delaware residents: 21% in 2014 and 40% in 2017.^30^ The CDC data suggests that these patterns were mainly caused by increasing flows traveling from Delaware to Pennsylvania and decreasing flows from Pennsylvania to Delaware. The current study focused on the resident abortion rate, a more appropriate evaluation outcome. Furthermore, pre‐post comparisons, such as those presented in the Upstream report, using a single pre‐period time point could easily mistake secular trends for program effects. Our study utilizes a well‐matched control group of non‐Delaware residents and a longer pre‐period to net out the secular changes in abortion that would have likely occurred in Delaware in the absence of the program.

## 2 METHODS

### 2.1 Data

We use abortion count data from the CDC's Abortion Surveillance System.^29^ The CDC annually collects abortion counts by the state of occurrence and the state of patients' residence. No within‐state subgroup information by state of the residence is provided. The data we use includes 46 states that consistently participate in the CDC Abortion Surveillance System from 2010 to 2019 (2019 is the most recent year of data, as of this writing). By 2019, all DelCAN payment policy changes and primary training activities had been implemented, and the media campaign had been fielded.^19^ The only intervention components remaining after 2019 were ongoing technical assistance activities. Because the vast majority of abortions occur by 12 weeks of pregnancy, we hypothesized that the effects on abortion would occur nearly contemporaneously with contraception effects, unlike effects on births.

Jurisdictions that were excluded due to incomplete data include California, the District of Columbia, Florida, Maryland, and New Hampshire. An important limitation of these data is that two jurisdictions neighboring Delaware (Maryland and the District of Columbia) are missing. Maryland did not report for all years, and the District of Columbia did not report for 2 years (2012 and 2016). This not only leads to those jurisdictions not being available as comparison states, but it also means that DE resident abortions occurring in those states are not counted. Our primary analyses exclude Maryland and DC, and we expect that that decision will not introduce a meaningful bias because they are excluded every year of the study period. Also, Maryland, DC, and Delaware all had stable abortion laws and a relatively stable number of abortion providers during the study period (DE: three clinics in 2014 and four in 2017; DC: five clinics in 2014 and 2017; MD: 25 clinics in 2014 and 2017).^31^, ^32^, ^33^ However, we conducted several sensitivity tests to explore the issue, which is discussed in more detail in the Appendix S1.

An alternative to the CDC's abortion counts are counts estimated by the Guttmacher Institute based on their own Abortion Provider Census. While Guttmacher's data is available for all states, it is only collected for two out of every 3 years. Several years are not available for our study period. We discuss our rationale for preferring the CDC estimates in more detail in the Appendix S1. We also show that, while we have less confidence in the Guttmacher data, we come to similar results using the Guttmacher counts instead of the CDC counts.

### 2.2 Outcome: Abortion rates

We use resident abortion rates, defined as the number of abortions per 1000 women aged 15 to 44. Population denominators were obtained from the US Census Bureau.^34^

### 2.3 Covariates

We obtained several state‐year covariates that previous studies have found to be associated with rates of unintended pregnancy and/or abortion.^13^, ^17^, ^32^, ^35^, ^36^, ^37^, ^38^, ^39^ First, we obtained socio‐demographic characteristics of each state‐year from the American Community Survey. The population characteristics describe the population of women of reproductive ages (15–44). These included age (% aged 15–24, 25–34, and 35–44), race (% non‐Hispanic White, non‐Hispanic Black, Hispanic, and non‐Hispanic other—including Asian, American Indian or Alaska Native, and Pacific Islander, and those who reported two or more races while not reporting Hispanic ethnicity), education (% those who had some college education, college graduate or more, and a high school degree or less), % noncitizen, % married, average number of children per household, insurance status (% of those who had public insurance, private insurance, and uninsured), average commute time to work (as a proxy for geographic access to services), median family income, % unemployed, and % population that is less than 100% of the federal poverty level.

We also obtained covariates describing health care supply which could affect the abortion rate through the available supply of health care professionals. From the Health Resources and Services Administrations, we obtained the per capita numbers of FQHCs, hospitals with obstetrics, obstetrics/gynecology physicians, and primary care physicians. From the Guttmacher Institute, we obtained the number of abortion clinics per capita and the percentage of women living in counties with no abortion provider.

We collected policy indicators that could have an important influence on abortion access and use: whether the state had expanded Medicaid in 2014 from the Kaiser Family Foundation and the abortion access index from the Guttmacher Institute.^31^, ^36^ Lastly, to account for policies not otherwise captured in the measures described above and to capture other socio‐political environment features that could influence abortion rates, we included congressional partisanship, which was measured as the percentage of Democrats in the state Senate and the percentage of Democrats in the state House from the National Council of State Legislatures.

### 2.4 Analysis

We used the synthetic control method (SCM) to measure the effect of DelCAN on the resident abortion rate.^40^ SCM form a control group, approximating what would have happened in Delaware had DelCAN not occurred by matching Delaware to a weighted combination of available control states. The weights are created using a data‐driven method that minimizes the difference in outcomes between Delaware and its “synthetic control” in the pre‐period (2010–2014). The effect of DelCAN is measured as the difference in the abortion rates in Delaware and the synthetic control in each post‐period year (2015–2019).

The optimization process used to generate the weights is based on pre‐period predictors that can include any combination of outcomes and covariates, from any combination of pre‐period years or their aggregates.^41^, ^42^, ^43^ Different sets of predictors have different advantages and disadvantages. Pre‐period outcomes from each pre‐period year maximize the amount of information about the object that is being matched, but can lead to overfitting.^40^, ^44^ Covariates that capture the causal factors influencing the outcome would avoid overfitting, but uncertainty about what the causal factors are and our ability to accurately observe them will degrade the quality of the match, relative to using outcomes. There are inherent limitations in combining covariates and outcomes as once all pre‐period year outcomes are included, there is no more predictive power to be had from covariates.^40^, ^44^

While flexibility in choosing predictor sets has advantages, it also presents the opportunity for cherry‐picking. To avoid that risk, we follow the guidance of Ferman et al. (2020) and present results from a pre‐defined set of models.^44^ We start with a base model that uses pre‐period outcomes from each pre‐period year. We then present the average of three alternative specifications using pre‐period outcomes from a subset of pre‐period years (2010, 2012, 2014; 2010, 2011, 2012; 2010, 2011, 2013, 2014) and the covariates that are explained above, which are averaged over the pre‐period. These alternative models help determine if the results from the base specification are robust enough to be reasonable alternatives in constructing the synthetic control weights. Following the pre‐defined plan outlined by Ferman et al. (2020) reduces the risk of cherry‐picking.^44^

We examine the quality of the match between Delaware and its synthetic control (i.e., the suitability of the synthetic control to represent what would have occurred in Delaware had the intervention not occurred) using a goodness‐of‐fit statistic, which is a pre‐treatment normalized mean squared error.^44^, ^45^

### 2.5 Sensitivity analyses

In addition to the alternative specifications and analyses using estimated abortion counts from Guttmacher, we conducted a series of other sensitivity tests (see the Appendix S1). We prefer to start our panel in 2010 because Delaware is missing from the CDC data in 2009. However, SCM tends to perform better with longer pre‐periods. We investigate if we come to similar conclusions when we extend the time series back to 2005 by adding 2005–2008. We examined if missing abortion counts in MD and DC for Delaware residents likely affected our findings by imputing abortion counts under various scenarios using the proportions of employed DE women working in neighboring states (DC, MD, NJ, and PA). In another sensitivity analysis, we excluded control states that implemented restrictive Title X funding policies on abortion (AZ, KS, MS, NE, OK, and WI)^46^ and states that had other contraceptive policy shocks, either to expand or restrict access (CO, IA, MO, SC, and TX).^11^, ^47^, ^48^, ^49^, ^50^, ^51^ Next, we conducted leave‐one‐out tests^42^ that re‐estimated the base model by excluding one state each time from the list of control states that received positive weight toward the synthetic control. This examines whether a confounding shock in a single control state explains our findings. We also examined if we came to different conclusions using different statistical procedures to estimate the synthetic control weights (penalized synthetic control).^52^

Across all models, statistical inferences are based on permutation tests.^41^, ^42^, ^43^, ^44^ The permutation test re‐estimates the synthetic control comparison using each of the 45 control states as a placebo treatment (i.e., 45 iterations). The *p*‐value is defined as the fraction of placebo states where the test statistic is at least as large as that observed in Delaware. The [BLINDED] IRB determined that this study did not meet the definition of human subjects research because it was based on publicly available, de‐identified data [reference # BLINDED].

## 3 RESULTS

**FIGURE 1:**
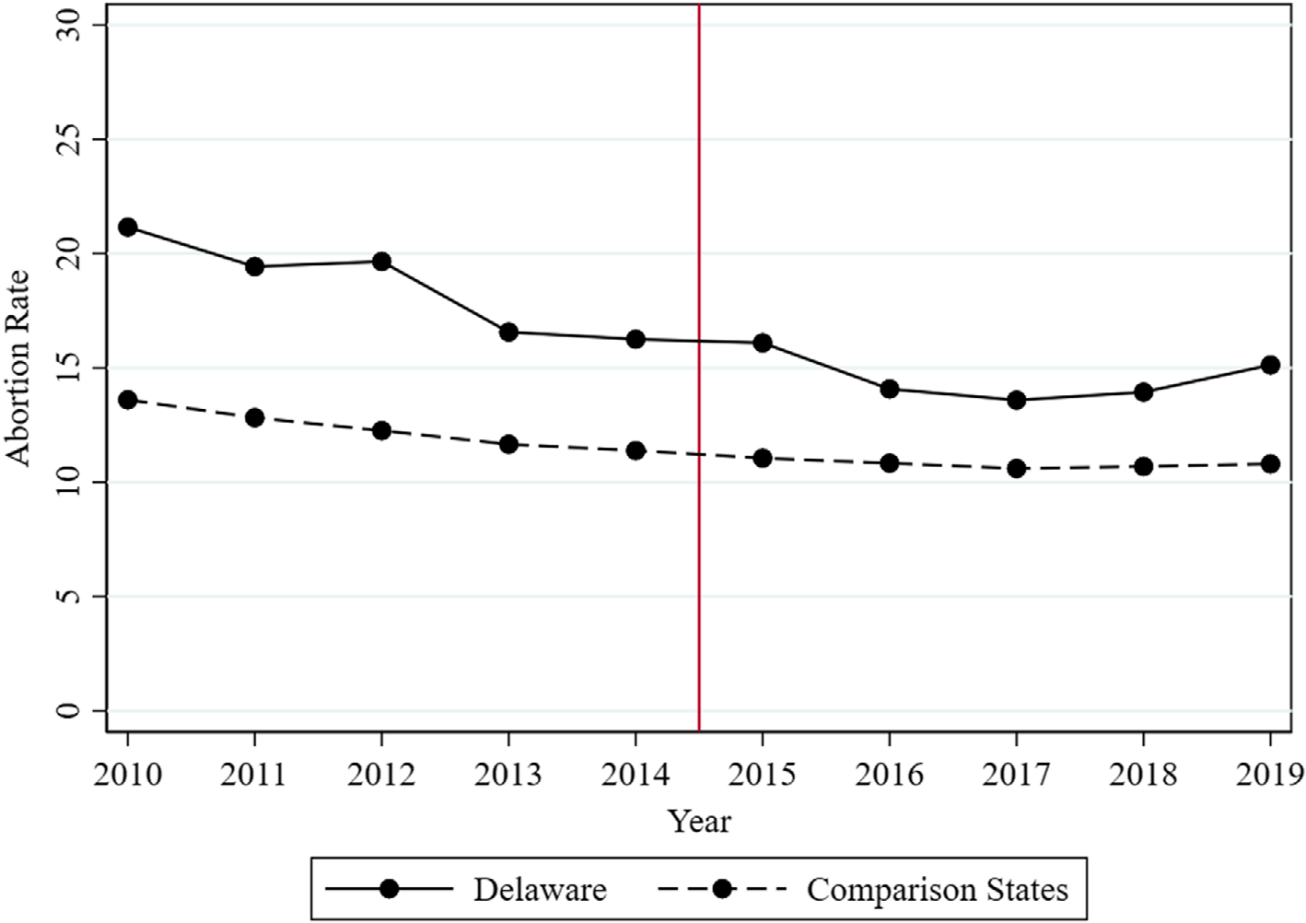
Resident abortion rates in delaware and the equally weighted average of potential comparison states. 2010–2019 CDC Abortion Surveillance data. Abortion rate = the number of abortions per 1000 women aged 15–44. The dashed line shows the average abortion rates in 45 comparison states. Four states (CA, FL, MD, and NH) and the District of Columbia are excluded due to missing data [Color figure can be viewed at wileyonlinelibrary.com]

Figure 1 shows unadjusted resident abortion rates in Delaware and the average rates of all other comparison states. Throughout the study period, Delaware had higher abortion rates than the average of the other 45 states (the rates are provided in Appendix Table A3). Delaware abortion rates declined from 21.2 in 2010 to 16.3 in 2014 (the last pre‐period year) and from 16.1 in 2015 to 15.1 in 2019. The Delaware abortion rate began to decline well before program implementation, suggesting that a pre‐post analysis within Delaware cannot be interpreted as the program's causal effect. Furthermore, the pre‐period decline was larger in Delaware than in the comparison states. This suggests comparing the change in Delaware to the change in the equally weighted average of the comparison states, as would be done in a difference‐in‐difference design, is also not appropriate.

**FIGURE 2:**
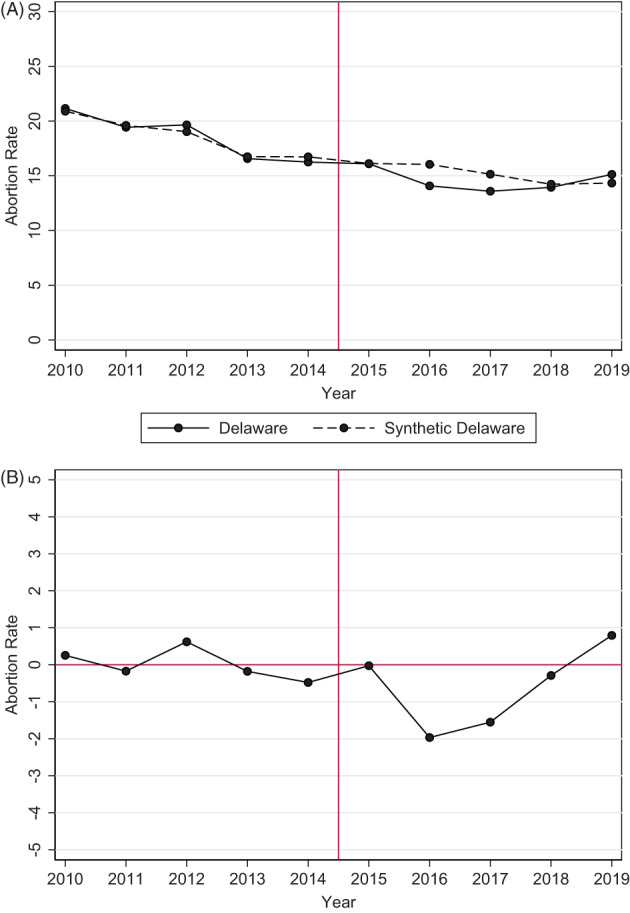
Resident abortion rates in delaware and synthetic delaware, 2010–2019. Panel a. Delaware versus Synthetic Delaware. Panel b. Differences of Abortion Rates. 2010–2019 CDC Abortion Surveillance data. Abortion rate = the number of abortions per 1000 women aged 15–44. The base model uses each pre‐period year outcome to generate the synthetic control unit. Four states (CA, FL, MD, and NH) and the District of Columbia are excluded due to missing data. Two states (HI and NY) contribute positive weight to synthetic Delaware. [Color figure can be viewed at wileyonlinelibrary.com]

Figure 2 plots the abortion rates in Delaware and synthetic Delaware (Panel A) and the difference between Delaware and synthetic Delaware (Panel B), as estimated by the base specification. The results in Figure 2 are summarized in Table 1. In contrast to the equally weighted average of other comparison states (Figure 1), pre‐treatment abortion rates in synthetic Delaware closely match Delaware in both their levels and their trends The goodness‐of‐fit of the synthetic control was 0.96 (Table 1), where greater than 0.8 is considered evidence of a well‐matched comparison group.^44^

**TABLE 1:** Summary of synthetic control method results.

	Average post‐period difference (Abortion rate = abortions/1000 women)	*p*‐value	Goodness‐of‐fit
Base model	−0.61	0.74	0.96
Average of alternative specifications	−1.22	0.59	0.79
Model using longer panel	−0.43	0.68	0.80

*Note*: Abortion rate = the number of abortions per 1000 women aged 15–44. The base model uses each pre‐period outcome to generate the synthetic control unit. Alternative specifications 1(2010, 2012, 2014), 2(2010, 2011, 2012), and 3(2010, 2011, 2013, 2014) vary based on sets of pre‐period outcomes but each adjusts for median family income, % age 15–24, % age 25–34, % non‐Hispanic White, % non‐Hispanic Black, % Hispanic, % some college education, % college or more education, % married, % unemployed, % <100% federal poverty level (FPL), % noncitizen, % public insurance, % private insurance, mean commute time, mean number of children per household, abortion accessibility index, per capita federally qualified health centers (FQHCs), per capita OB/GYN physicians, per capita primary care physician (PCP), % women with no abortion provider, whether expanded Medicaid in 2014, % Democrats in state Senate, and % Democrats in state House The model using longer panel used data of 2005–2019 (except for 2009 due to missing data). *p*‐values come from permutations tests. Goodness‐of‐fit is a measure of the quality of the match and is defined as pre‐treatment normalized mean squared error. Four states (CA, FL, MD, NH) and the District of Columbia are excluded due to missing data.

Source: 2010–2019 CDC Abortion Surveillance data.

Figure 2 suggests that Delaware experienced some decreases in abortion rates in 2016 and 2017 compared with synthetic Delaware. However, the effects attenuated in 2018 and 2019 such that the abortion rates again became about the same or slightly higher in Delaware, compared to synthetic Delaware. In the post‐intervention period (2015–2019), Delaware had on average 0.61 fewer abortions per 1000 women compared with synthetic Delaware (Table 1), but the point estimate was not statistically significant (*p* = 0.71). The average of the alternative specifications had a similar but slightly larger effect—1.22 fewer abortions on average per 1000 women ages 15 to 44 in Delaware than synthetic Delaware—but was also not statistically significant (*p* = 0.59) (Table 1). Plots describing the alternative specifications are available in Appendix Figure A1. Of particular note, the average goodness‐of‐fit from the alternative specifications was lower than our preferred model (0.79). While the alternatives are substantially similar to the base model, the worse goodness‐of‐fit statistic suggests less confidence should be placed in it.

In the base model, the weights of synthetic Delaware are 0.422 for Hawaii and 0.579 for New York (Appendix Table A1). Sparsity in the weights is a common and desired feature of well‐matched synthetic control units.^40^ The pre‐period balance is shown in Table 2.

**TABLE 2:** Predictor balance by specification.

		Synthetic control			
Variables	Delaware	Base model	Alternative 1	Alternative 2	Alternative 3
Pre‐period abortion rates					
2010	21.16	20.90	20.71	20.61	20.42
2011	19.43	19.60		19.90	19.26
2012	19.66	19.04	19.27	19.36	
2013	16.57	16.75			17.09
2014	16.26	16.73	17.17		16.74
Covariates (pre‐period average)					
Median family income	$ 59,540	$ 63,012	$ 55,751	$ 54,403	$ 52,934
% age 15–24	32.7%	31.2%	32.0%	32.1%	32.0%
% age25‐34	33.6%	35.5%	34.7%	34.5%	34.6%
% non‐Hispanic White	61.3%	39.4%	59.8%	57.9%	59.1%
% non‐Hispanic Black	25.1%	10.2%	17.1%	21.4%	19.3%
% Hispanic	6.6%	14.1%	11.9%	11.2%	11.1%
% some college educ	30.4%	31.1%	29.9%	30.7%	31.1%
% college or more	27.6%	31.6%	30.9%	30.7%	29.0%
% married	36.2%	38.4%	37.3%	35.9%	37.3%
% unemployed	6.7%	6.1%	7.0%	7.6%	7.3%
% <100FPL	16.2%	16.6%	19.3%	20.5%	20.3%
% noncitizen	7.9%	13.5%	13.6%	11.5%	12.1%
% public insurance	24.1%	21.2%	20.1%	22.1%	19.6%
% private insurance	65.2%	67.4%	61.7%	61.6%	61.1%
Mean commute time	23.6	28.8	27.8	28.0	26.7
Mean number of children per household	0.94	0.86	0.93	0.92	0.93
Abortion hostile index	2.0	0.6	2.5	2.7	2.7
Per capita FQHC	0.000112	0.000204	0.000152	0.000221	0.000165
Per capita hospitals with OB	0.000047	0.000060	0.000072	0.000079	0.000069
Per capita OBGYN	0.000716	0.001183	0.001053	0.001058	0.001035
Per capita primary care physician	0.007295	0.008425	0.007483	0.007484	0.007349
Per capita abortion provider	0.000039	0.000081	0.000034	0.000034	0.000034
% women with no provider	18.0%	4.0%	22.5%	30.4%	26.2%
Expanded Medicaid in 2014	1.0	1	0.8	0.8	0.6
% Dem. in state Senate	65.7%	69.3%	51.9%	53.1%	46.7%
% Dem. in state House	63.4%	75.9%	58.5%	62.3%	55.1%

*Note*: Abortion rate = number of abortions per 1000 women ages 15–44. The base model uses each pre‐period year outcome (2010–2014) to generate the synthetic control unit. The base model uses each pre‐period outcome to generate the synthetic control unit. Alternative specifications 1(2010, 2012, 2014), 2(2010, 2011, 2012), and 3(2010, 2011, 2013, 2014) vary based on sets of pre‐period outcomes but each adjusts for covariates listed in Column 1. Four states (CA, FL, MD, NH) and the District of Columbia are excluded due to missing data. The italicized cells in the second column (covariates) are provided as additional information, although the covariates were not included in estimating the base model.

Source: 2010–2014 CDC Abortion Surveillance data.

### 3.1 Robustness tests

**FIGURE 3:**
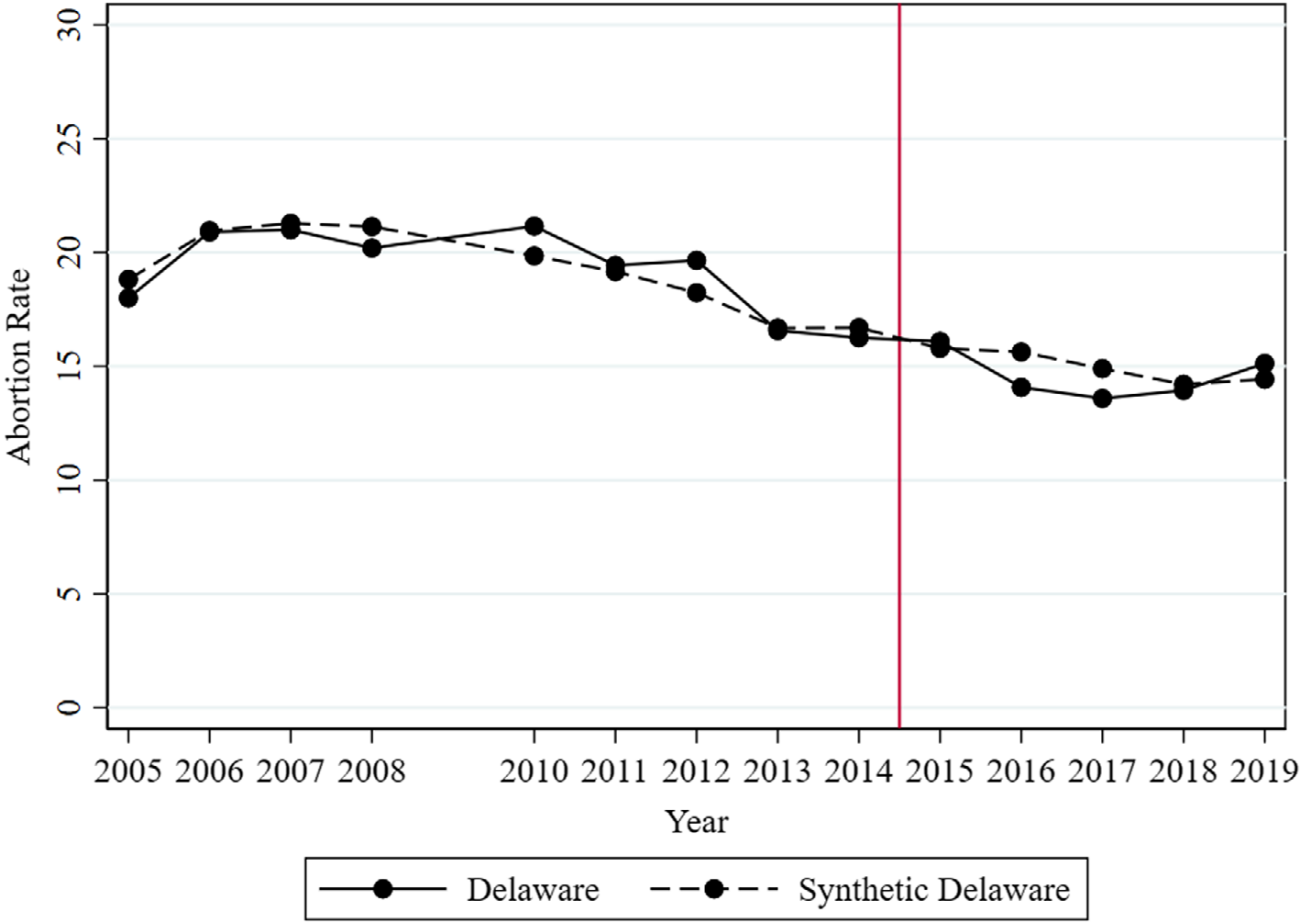
Robustness Test Using a Longer Pre‐Period, 2005–2019. 2005–2019 CDC Abortion Surveillance data. Abortion rate = number of abortions per 1000 women ages 15–44. The model used base model with each pre‐period year outcome (2005–2008 and 2010–2014) to generate the synthetic control unit. Six states (CA, FL, KY, LA, MD, and NH) and the District of Columbia are excluded due to missing data. Four states (AZ, CT, IL, and NY) contribute positive weight to synthetic Delaware. The year of 2009 was omitted because Delaware did not report abortion data to CDC in 2009. [Color figure can be viewed at wileyonlinelibrary.com]

Figure 3 displays resident abortion rates in Delaware versus synthetic Delaware using the longer panel that started in 2005, and the results from this model are summarized in Table 1. We come to the same conclusions using the longer panel. The Appendix S1 reports the remaining robustness tests. While the match obtained from the Guttmacher data was considerably worse than that obtained from the CDC, the Guttmacher data suggests the same conclusions regardless of using the same donor pool as in the CDC analysis or using all 50 states and DC as a donor pool. Imputing abortion counts occurring in MD and DC to Delaware residents and re‐examining our models did not change our findings. Excluding states that experienced policy shocks potentially affecting abortion, the leave‐one‐out test, and using an alternative estimator for the synthetic control weights, all suggested similar results to those summarized in Table 1. Our results might mask heterogeneous effects across groups. Unfortunately, the CDC data lacks resident abortion rates by subgroup. However, in the Appendix S1, we show available abortion statistics by age group, produced by the Delaware Division of Public Health. While the trend was noisier, it was generally parallel for older and younger populations, suggesting that treatment effects were likely similar by age, but this is only a suggestive pattern.^53^ Similar subgroup analyses by other characteristics are not possible because the data are not collected (e.g., poverty) or group definitions change over time (e.g., race).

## 4 DISCUSSION

Using CDC Abortion Surveillance data and the SCM, this paper examined whether a comprehensive contraceptive access initiative in Delaware affected resident abortion rates. We did not find consistent evidence that the intervention decreased abortions. We did observe that Delaware experienced larger declines in abortion compared with the synthetic control in the early years of the intervention. However, even in the year with the largest observed difference, the magnitude (1.97 fewer abortions per 1000 women ages 15–44 or a 10.6% reduction from the average pre‐period rate) was much smaller than the pre‐post estimate suggested by a previous report^27^ and was consistent with the random variation one would expect in the absence of an effect. By 2019, the magnitude was nearly zero.

Previous studies suggest mixed evidence on the effects of other comprehensive programs on abortion. For example, Biggs et al. (2015) found that increased LARC uptake after the Iowa Initiative to Reduce Unintended Pregnancies was associated with decreased abortion rates.^17^ However, the study was based on measuring the relationship between region‐level LARC use rates and abortion rates, it lacked a clear comparison group, and the initiative co‐occurred with an expansion of abortion services via telehealth. These challenges create substantial uncertainty about the effect of the Iowa program on abortion. Our results would also seem to disagree with the reduced abortions observed in the CHOICE Program.^10^ However, the CHOICE intervention cohort only included participants who had a desire to change to reversible contraceptive methods. The abortion experience of this cohort was compared against that of regional and national populations that were not equally selected.^10^ It is likely that the experience of the CHOICE cohort is not generalizable, and there remains uncertainty if the cohort would have experienced reductions in abortion in the absence of the intervention.

Our methods and results are more in line with Kelly et al.'s (2020) that investigated the effects of the Colorado Family Planning Initiative on abortions using difference‐in‐differences analyses.^13^ They did not find definitive evidence that the program reduced abortions. While they did find significant effects in some post‐treatment years among women ages 15 to 19 (but no effects among older groups), they also found disparate trends in the pre‐treatment period in Colorado versus the comparison jurisdictions, which complicates interpreting their results as causal.

Given the limitations of the literature, there remains uncertainty about whether the null results that we obtained in Delaware should be thought of as a common feature of all similar coordinated programs or if the DelCAN experience was unique. However, our failure to find evidence of an effect using a rigorous approach and population representative data does suggest programs like DelCAN might be unlikely to reduce unintended pregnancies that result in abortion. This is useful information for other states considering DelCAN‐style programs. However, program designers should also consider the fact that the effect of programs like DelCAN on outcomes, such as abortion is likely to vary considerably across geography as a function of health system resources, abortion access environments, and travel distances to neighboring jurisdictions. Furthermore, program designers should also recognize that there is considerable value in increasing access to high‐quality, person‐centered contraceptive care, even if that care does not alter unintended pregnancy in the population.

There are several possible explanations for why we did not observe strong evidence of an effect on abortion from DelCAN, despite previous evidence that the program increased LARC use. First, we expected that effects on abortion would be contemporaneous with changes in contraceptive use observed in other studies.^23^, ^24^, ^25^ However, program effects in Delaware may grow over time as providers continue to adapt to new practice styles and patients become more aware of available services, especially considering that the program components were gradually introduced over 2015–2020. Furthermore, long‐acting methods provide sustained protection, so pregnancy protection should accumulate in the population over time. Additional study is warranted to examine the program's effects over longer time horizons.

Second, the uptake of more effective contraceptives from DelCAN^23^, ^24^, ^25^, ^26^, ^54^ could have been by people who would have, in the absence of the program, not chosen abortion, by people who substituted more effective methods for less effective methods, or by people who would have taken other precautionary actions (such as reducing sexual activity) had they not had access to the contraceptive services facilitated by DelCAN. Boudreaux et al. (2020) found suggestive evidence that increased LARC use among Title X patients was partly due to substitution for sterilization; however, existing work has struggled to find clear and consistent evidence about changes to other method types.^23^

Third, we were unable to examine effects by age, but previous studies suggest that comprehensive initiatives like DelCAN might have larger effects on younger patients. For example, despite potential confounding from pre‐existing disparate trends, evaluations from Colorado provide suggestive evidence of abortion effects in patients under the age of 20.^13^ Other studies on DelCAN also found larger effects on LARC uptake among teenagers.^24^, ^25^ However, as we show in Appendix Figure A4, the trend in abortion rates within Delaware was generally similar for younger and older patients, which is not suggestive of heterogeneous effects by age. Furthermore, while uncovering subgroup effects remains an important goal, the intervention sought to reduce unintended pregnancy for all persons in Delaware at risk for unintended pregnancy, a goal that population‐wide rates align with.

It is also possible that DelCAN's effect on contraceptive use was not substantial enough to meaningfully alter abortion rates. Regardless of the underlying reasons, our results are important to stakeholders in Delaware and to other states that are engaged in similar initiatives.^1^, ^18^

There is not a strong public health case for policymakers to prefer contraceptives over abortion as a tool for reducing the risk of unintended birth. Both are safe and effective and reduce the adverse consequences associated with unintended birth.^3^, ^4^, ^55^, ^56^, ^57^ Nonetheless, abortion rates are a useful evaluation metric because the availability of high‐quality data on state‐year unintended pregnancies is limited. The National Survey of Family Growth is the only survey capable of producing estimates of unintended pregnancy, but its sample is too small to generate state‐level estimates. Abortion is a useful proxy given that nearly all abortions are from unintended pregnancies and a substantial fraction (42%) of unintended pregnancies result in abortion.^1^, ^18^ Furthermore, while the public health case for preferring contraceptives over abortions is weak, the policy case might not be. The increasingly restrictive abortion environment will likely lead policymakers, providers, and advocates to prioritize contraceptive access.

This study has limitations. First, while the CDC data allows us to track abortion rates over time across nearly every state, it is subject to measurement error. As reporting abortion statistics is not federally mandated, states have varying abortion reporting requirements, which might result in underreporting.^58^, ^59^ However, if that error is consistent within states and across years, it should not seriously bias our results. We also investigated the feasibility of using Guttmacher's abortion data, which is based on their own surveys of abortion providers.^60^ However, that data are not collected for several years during our study period.^60^ While we prefer the CDC estimates relative to Guttmacher's, we show in the Appendix S1 that we come to similar results using the Guttmacher data. Second, our findings may not be generalizable to other states that have different health systems and social contexts. Finally, our study design rested on the untestable assumption that the trend in synthetic control represented what would have happened in Delaware had the intervention not occurred. While violations of that assumption are an important concern, our preferred model produced a strong match to Delaware, and we came to consistent results across a range of alternative modeling assumptions.

## 5 CONCLUSIONS

Our results do not provide strong evidence of an effect of DelCAN on abortion. This does not rule out that the program had important benefits by easing barriers to contraceptive methods or by reducing unintended pregnancies that would have resulted in births. Nonetheless, our results suggest that through 2019, the program did not have a meaningful impact on the 40% of unintended pregnancies that result in abortion. Our results should inform other states and organizations engaged in improving access to contraceptive care.

## Supporting information


**Appendix S1.** Supporting Information.
