# Supplementary material for: Contraceptive access reform and abortion: Evidence from Delaware
Source: Health Serv Res. 2023 Apr 9;58(4):781–91. doi: 10.1111/1475-6773.14156 (PMC10315387; doi:10.1111/1475-6773.14156)
Supplement: Supplementary file 1 — Appendix S1. Supporting Information. [file HESR-58-781-s001.docx]

**Contraceptive Access Reform and Abortion: Evidence from Delaware: Appendix**

**Content**

Appendix A. Supplemental Tables and Figures

Appendix B. Sensitivity Analysis

B.1. Leave-one-out test

B.2. Robustness test using stable donor pool

B.3. Using an alternative approach to calculating synthetic control weights: Penalized synthetic control

B.4. Handling missing Maryland and DC abortion counts by Delaware residents

Appendix C. Abortion Data from the CDC and Guttmacher Institute

**Appendix A. Supplemental Tables and Figures**

This section presents tables and figures on the synthetic control results that are supplementary to our main document. Appendix Table A1 shows the weights of each state to synthetic Delaware in the base model and the alternative specifications.

Appendix Table A1. Synthetic Control Weights

|  |  | Alternative specification | | |  |  | Alternative specification | | |
| --- | --- | --- | --- | --- | --- | --- | --- | --- | --- |
| State | Base | 1 | 2 | 3 | State | Base | 1 | 2 | 3 |
| Alabama | 0 | 0 | 0 | 0 | Nevada | 0 | 0 | 0 | 0 |
| Alaska | 0 | 0 | 0 | 0 | New Jersey | 0 | 0 | 0 | 0 |
| Arizona | 0 | 0 | 0 | 0 | New Mexico | 0 | 0 | 0 | 0 |
| Arkansas | 0 | 0 | 0 | 0 | New York | 0.578 | 0.477 | 0.523 | 0.475 |
| Colorado | 0 | 0 | 0 | 0 | North Carolina | 0 | 0 | 0 | 0 |
| Connecticut | 0 | 0 | 0 | 0 | North Dakota | 0 | 0 | 0 | 0 |
| Georgia | 0 | 0 | 0 | 0 | Ohio | 0 | 0 | 0 | 0 |
| Hawaii | 0.422 | 0.005 | 0 | 0 | Oklahoma | 0 | 0 | 0 | 0 |
| Idaho | 0 | 0 | 0 | 0 | Oregon | 0 | 0 | 0 | 0 |
| Illinois | 0 | 0.291 | 0.291 | 0 | Pennsylvania | 0 | 0 | 0 | 0 |
| Indiana | 0 | 0 | 0 | 0 | Rhode Island | 0 | 0 | 0 | 0 |
| Iowa | 0 | 0 | 0.017 | 0 | South Carolina | 0 | 0 | 0 | 0.251 |
| Kansas | 0 | 0 | 0 | 0 | South Dakota | 0 | 0 | 0 | 0 |
| Kentucky | 0 | 0 | 0 | 0 | Tennessee | 0 | 0 | 0 | 0 |
| Louisiana | 0 | 0 | 0 | 0 | Texas | 0 | 0.198 | 0 | 0.154 |
| Maine | 0 | 0 | 0 | 0 | Utah | 0 | 0 | 0 | 0 |
| Massachusetts | 0 | 0 | 0 | 0 | Vermont | 0 | 0 | 0 | 0 |
| Michigan | 0 | 0 | 0 | 0 | Virginia | 0 | 0 | 0 | 0 |
| Minnesota | 0 | 0 | 0 | 0 | Washington | 0 | 0 | 0 | 0.121 |
| Mississippi | 0 | 0 | 0.169 | 0 | West Virginia | 0 | 0 | 0 | 0 |
| Missouri | 0 | 0 | 0 | 0 | Wisconsin | 0 | 0 | 0 | 0 |
| Montana | 0 | 0.029 | 0 | 0 | Wyoming | 0 | 0 | 0 | 0 |
| Nebraska | 0 | 0 | 0 | 0 | . | . | . | . | . |

**Source**: 2010-2019 CDC Abortion Surveillance data. **Notes**: The base model uses each pre-period year outcome (2010-2014) to generate the synthetic control unit. Alternative specifications 1(2010, 2012, 2014), 2(2010, 2011, 2012), and 3(2010, 2011, 2013, 2014) vary based on sets of pre-period outcomes but each adjusts for covariates including median family income, % age 15-24, % age 25-34, % non-Hispanic White, % non-Hispanic Black, % Hispanic, % some college education, % college or more education, % married, % unemployed, % <100% Federal Poverty Level (FPL), % noncitizen, % public insurance, % private insurance, mean commute time, mean number of children per household, abortion accessibility index, per capita federally qualified health centers (FQHCs), per capita OB/GYN physicians, per capita primary care physician (PCP), % women with no abortion provider, whether expanded Medicaid in 2014, % Democrats in state Senate, and % Democrats in state House. Four states (CA, FL, MD, NH) and the District of Columbia are excluded due to missing data.

Appendix Figure A1 shows the trends in abortion rates of synthetic Delaware for all the three alternative specifications that used subsets of pre-period outcomes and covariates. The estimated abortion rates are shown in Appendix Table A2.

| Appendix Figure A1. Base Model and Three Alternative Specifications |
| --- |
| 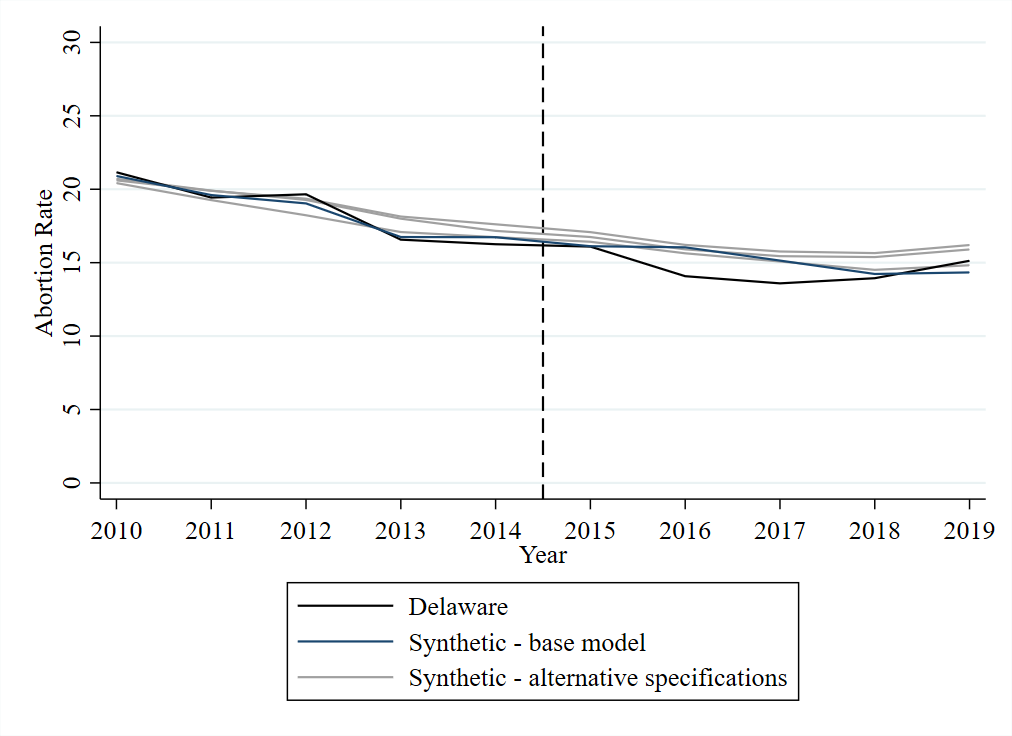 |
| **Source**: 2010-2019 CDC Abortion Surveillance data. **Notes**: Abortion rate = number of abortions per 1,000 women ages 15-44. The base model uses each pre-period year outcome (2010-2014) to generate the synthetic control unit. Alternative specifications 1(2010, 2012, 2014), 2(2010, 2011, 2012), and 3(2010, 2011, 2013, 2014) vary based on sets of pre-period outcomes but each adjusts for covariates including median family income, % age 15-24, % age 25-34, % non-Hispanic White, % non-Hispanic Black, % Hispanic, % some college education, % college or more education, % married, % unemployed, % <100% Federal Poverty Level (FPL), % noncitizen, % public insurance, % private insurance, mean commute time, mean number of children per household, abortion accessibility index, per capita federally qualified health centers (FQHCs), per capita OB/GYN physicians, per capita primary care physician (PCP), % women with no abortion provider, whether expanded Medicaid in 2014, % Democrats in state Senate, and % Democrats in state House. |

Appendix Table A2. Pre- and Post-Period Results on Abortion Rates for Each Specification

| Year | Delaware | Simple average | Synthetic Control | | | |
| --- | --- | --- | --- | --- | --- | --- |
|  |  | Comparison states | Base Model | Alternative 1 | Alternative 2 | Alternative 3 |
| 2010 | 21.2 | 13.6 | 20.9 | 20.7 | 20.6 | 20.4 |
| 2011 | 19.4 | 12.8 | 19.6 | 19.9 | 19.9 | 19.3 |
| 2012 | 19.7 | 12.3 | 19.0 | 19.3 | 19.4 | 18.2 |
| 2013 | 16.6 | 11.7 | 16.7 | 18.0 | 18.1 | 17.1 |
| 2014 | 16.3 | 11.4 | 16.7 | 17.2 | 17.6 | 16.7 |
| **2015** | 16.1 | 11.1 | 16.1 | 16.7 | 17.1 | 16.4 |
| **2016** | 14.1 | 10.8 | 16.0 | 15.9 | 16.2 | 15.6 |
| **2017** | 13.6 | 10.6 | 15.1 | 15.4 | 15.8 | 15.1 |
| **2018** | 13.9 | 10.7 | 14.2 | 15.4 | 15.7 | 14.5 |
| **2019** | 15.1 | 10.8 | 14.3 | 15.9 | 16.2 | 14.8 |

**Source**: 2010-2019 CDC Abortion Surveillance data. **Notes**: Abortion rate = number of abortions per 1,000 women ages 15-44. The base model uses each pre-period year outcome (2010-2014) to generate the synthetic control unit. Alternative specifications 1(2010, 2012, 2014), 2(2010, 2011, 2012), and 3(2010, 2011, 2013, 2014) vary based on sets of pre-period outcomes but each adjusts for covariates including median family income, % age 15-24, % age 25-34, % non-Hispanic White, % non-Hispanic Black, % Hispanic, % some college education, % college or more education, % married, % unemployed, % <100% Federal Poverty Level (FPL), % noncitizen, % public insurance, % private insurance, mean commute time, mean number of children per household, abortion accessibility index, per capita federally qualified health centers (FQHCs), per capita OB/GYN physicians, per capita primary care physician (PCP), % women with no abortion provider, whether expanded Medicaid in 2014, % Democrats in state Senate, and % Democrats in state House. Four states (CA, FL, MD, NH) and the District of Columbia are excluded due to missing data.

Appendix Figure A4 and Appendix Table A3 show abortion rates among teenage women (ages 15-19) and adult women (ages 20-44) had similar trends among Delaware residents during 2014-2019. This suggests that the effects of DelCAN were likely not heterogeneous by age. Note that the abortion rates for this graph are from Delaware Vital Statistics Annual Report (Delaware Health Statistics Center, 2019), and as a result, they are different from the abortion rates from the CDC Surveillance data, which is used for our main analysis. The age-stratified data are only publicly available starting in 2014.

Appendix Figure A4. Abortion Rates by Age Among Delaware Residents


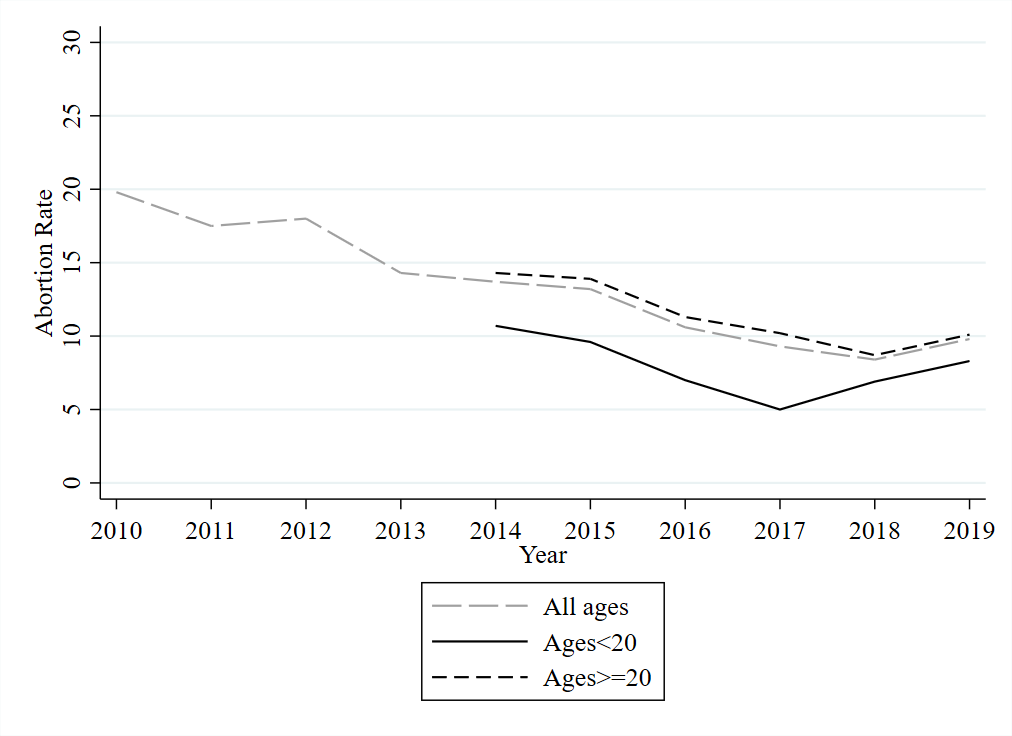


**Source**: Delaware Division of Public Health Vital Statistics Annual Report 2010-2018. **Notes**: Abortion rate = number of abortions per 1,000 women.

Appendix Table A3. Abortion Rates by Age Among Delaware Residents

| Year | All Ages | Ages <20 | Ages >=20 |
| --- | --- | --- | --- |
| 2010 | 19.8 | . | . |
| 2011 | 17.5 | . | . |
| 2012 | 18.0 | . | . |
| 2013 | 14.3 | . | . |
| 2014 | 13.7 | 10.7 | 14.3 |
| 2015 | 13.2 | 9.6 | 13.9 |
| 2016 | 10.6 | 7.0 | 11.3 |
| 2017 | 9.3 | 5.0 | 10.2 |
| 2018 | 8.4 | 6.9 | 8.7 |
| 2019 | 9.8 | 8.3 | 10.1 |

**Source**: Delaware Division of Public Health Vital Statistics Annual Report 2010-2019 and Census Bureau population estimates 2010-2019. **Notes**: Abortion rate = number of abortions per 1,000 women.

**Appendix B. Sensitivity Analysis**

*B.1. Leave-one-out test*

Appendix Figure B1-1 shows the synthetic control results of the leave-one-out test. The leave-one-out test is to perform permuted synthetic controls by leaving each unit out of the donor states that had positive weights and examine whether taking one unit out from the control group critically affects the synthetic control result. The leave-one-out tests were conducted on HI and NY with base model, on HI, IL, MT, NY, and TX with alternative specification 1, on IA, IL, MI, and NY with alternative specification 2, and on NY, SC, TN, and WA with alternative specification 3.

The leave-one-out tests on the four models, except for when leaving out NY, did not have significant differences in creating synthetic Delaware (solid gray lines) from the original models (black dashed line). The outlier synthetic, which has constantly lower abortion rates, is when excluding the state of New York from the control group (gray dashed line). This is because Delaware has the second-highest pre-period average abortion rates among the 46 states, where New York is the first-ranked. That is, without New York in the control group, it actually violates the assumption of synthetic control that the vector of the pre-period predictors of the treatment unit belongs to the convex hull of the vectors of the pre-period predictors of the control units (Abadie et al., 2010). Thus, the control group without New York essentially cannot match the pre-period outcomes of Delaware. However, even though the levels of abortion rates are constantly lower than the true Delaware (and all other models), the trends of the leave-NY-out models are not different from the original model or the rest of the leave-out-out permutations. Thus, it does not invalidate our findings on the DelCAN effects on the abortion rates. In sum, New York was an exception as it was the only state that has higher pre-period abortion rates than Delaware, and for other states, taking out one state from the donor pool did not change the synthetic control results.

| Appendix Figure B1-1. Leave-One-Out Test | |
| --- | --- |
| Panel a. Base model | Panel b. Alternative specification 1 |
| 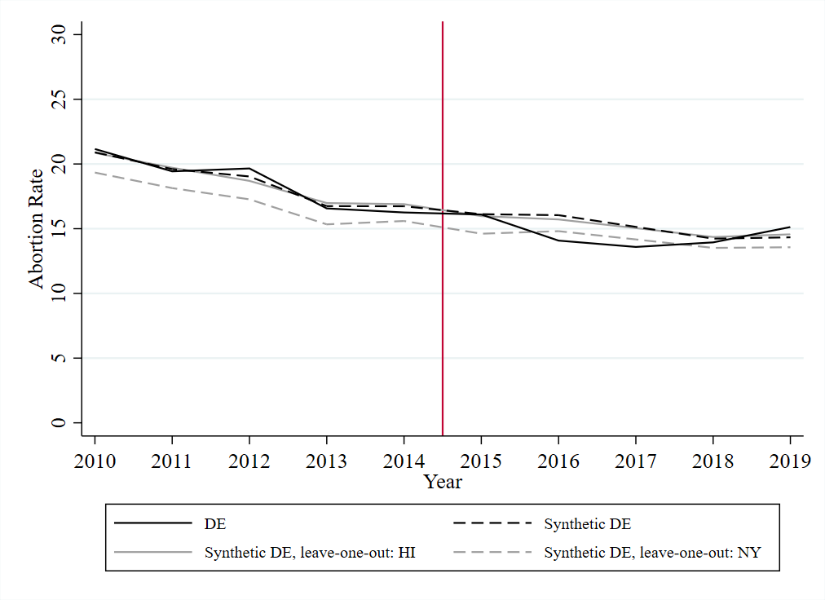 | 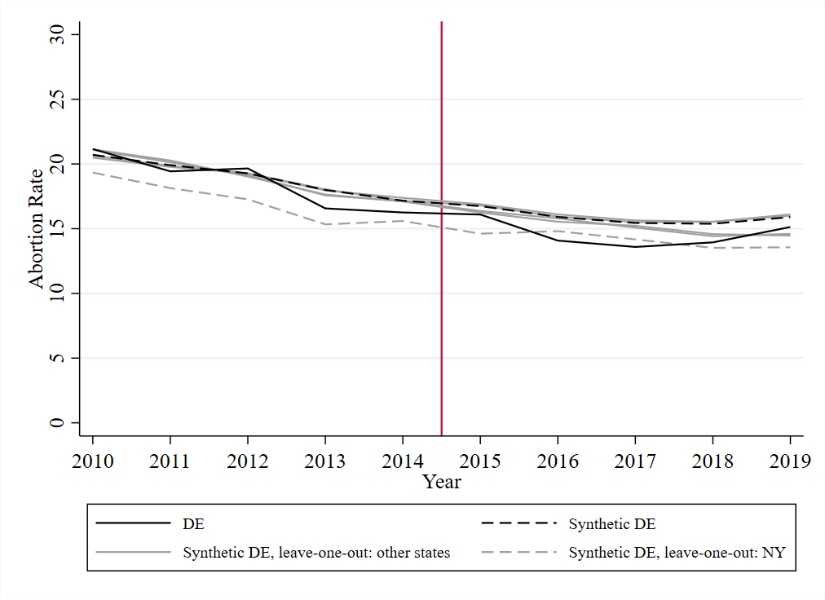 |
| Panel c. Alternative specification 2 | Panel d. Alternative specification 3 |
| 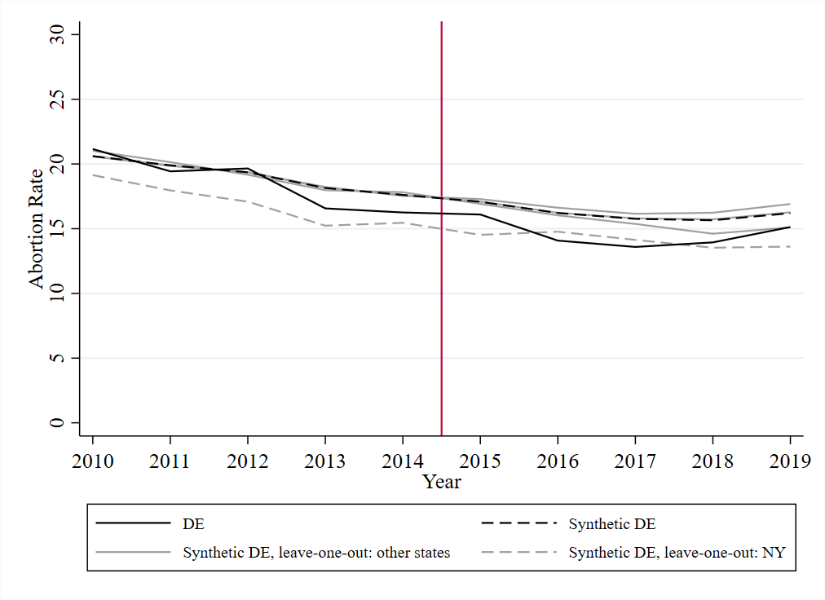 | 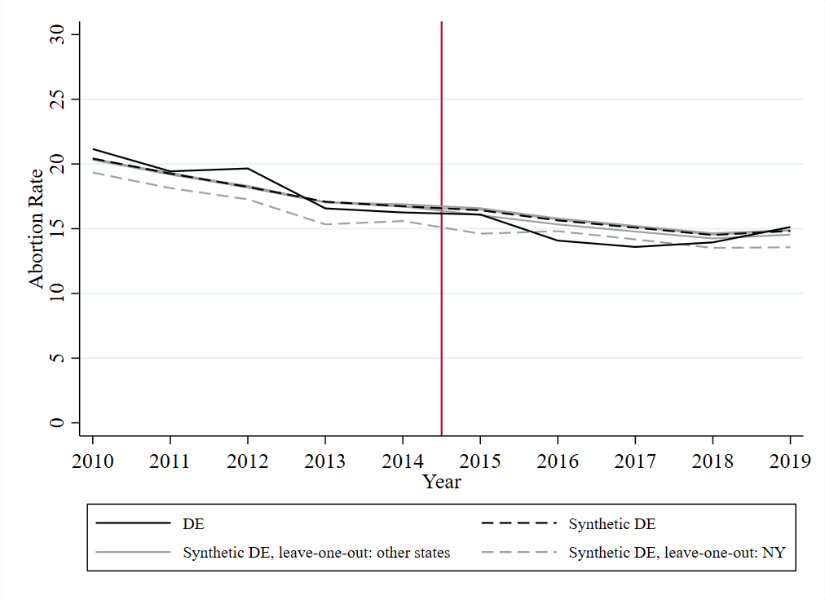 |
| **Source**: 2010-2019 CDC Abortion Surveillance data. **Notes**: Abortion rate = number of abortions per 1,000 women ages 15-44.  The base model uses each pre-period year outcome (2010-2014) to generate the synthetic control unit. The alternative specifications use alternative sets of pre-period outcomes in combination with covariates in each pre-period year. | |

*B.2. Robustness test using stable donor pool*

As another sensitivity analysis, we conducted sensitivity analyses excluding two sets of states. First, we excluded states that have restrictive Title X funding policies (Arizona, Kansas, Mississippi, Nebraska, Oklahoma, and Wisconsin) (Guttmacher Institute, 2022). Second, we excluded states that had policy shocks that affect contraceptive access before or during the study period (Colorado, Iowa, Missouri, South Carolina, and Texas) (Colorado Department of Public Health and Environment, 2017; McNicholas et al., 2014; Philliber Research Associates, 2010; Sundstrom et al., 2016, 2019; White et al., 2015).

The synthetic control results using a stable donor pool are shown in Appendix Figure B2-1 and Appendix Table B2-1. The weights are displayed in Appendix Table B2-2. The synthetic control result with stable state donors does not much differ from the main result. The base model with the stable donor pool had exactly the same result as the base model in our main text. The three alternative specifications after restricting donor states that experienced policy shocks (average effects: -1.04 abortions per 1,000 women, p=0.60) had similar results as our alternative specifications in the primary analysis (average effects: -1.22 abortions per 1,000 women, p=0.59).

| Appendix Figure B2-1. Base Model and Three Alternative Specifications with Stable States Donor Pool |
| --- |
| 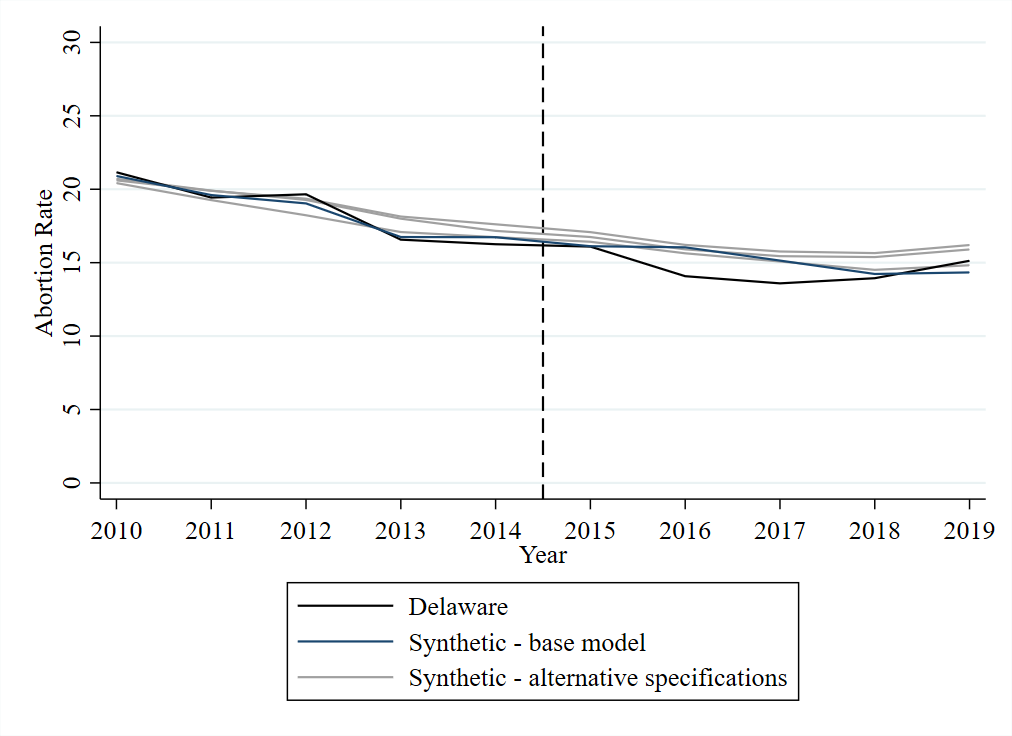 |
| **Source**: 2010-2019 CDC Abortion Surveillance data. **Notes**: Abortion rate = number of abortions per 1,000 women ages 15-44. The base model uses each pre-period year outcome (2010-2014) to generate the synthetic control unit. The alternative specifications use alternative sets of pre-period outcomes in combination with covariates in each pre-period year. 34 states are included in the synthetic control donor pool. Four states (CA, FL, MD, NH) and the District of Columbia are excluded due to missing data. Additionally, in this robustness test, six states (AZ, KS, MS, NE, OK, WI) are excluded as they have restrictive Title X funding policies, and five states (CO, IA, MO, SC, TX) are excluded as they had policy shocks that affect contraceptive access before or during the study period. |

| Appendix Table B2-1. Summary of Synthetic Control Method Results with Stable Donor Pool | | | | |
| --- | --- | --- | --- | --- |
|  |  | Average Post-Period Difference | P-Value | Goodness-of-fit |
|  |  |  |  |  |
| Base Model |  | -0.61 | 0.71 | 0.96 |
| Average of Alternative Specifications | | -1.04 | 0.60 | 0.77 |
| **Source**: 2010-2019 CDC Abortion Surveillance data. **Notes**: Abortion rate = the number of abortions per 1,000 women aged 15-44. The base model uses each pre-period year outcome to generate the synthetic control unit. The alternative specifications use alternative sets of pre-period outcomes in combination with covariates in each pre-period year. P-values come from permutations tests. Goodness-of-fit is a measure of the quality of the match and is defined as pre-treatment normalized mean squared error. 34 states are included in the synthetic control donor pool. Four states (CA, FL, MD, NH) and the District of Columbia are excluded due to missing data. Additionally, in this robustness test, six states (AZ, KS, MS, NE, OK, WI) are excluded as they have restrictive Title X funding policies, and five states (CO, IA, MO, SC, TX) are excluded as they had policy shocks that affect contraceptive access before or during the study period. | | | | |
|  | | | | |

Appendix Table B2-2. Synthetic Control Weights with Stable Donor Pool

|  |  | Alternative specification | | |  |  | Alternative specification | | |
| --- | --- | --- | --- | --- | --- | --- | --- | --- | --- |
| State | Base | 1 | 2 | 3 | State | Base | 1 | 2 | 3 |
| Alabama | 0 | 0 | 0.053 | 0.249 | New Jersey | 0 | 0 | 0 | 0 |
| Alaska | 0 | 0 | 0.055 | 0 | New Mexico | 0 | 0 | 0 | 0 |
| Arkansas | 0 | 0 | 0 | 0 | New York | 0.578 | 0.539 | 0.48 | 0.5 |
| Connecticut | 0 | 0 | 0 | 0 | North Carolina | 0 | 0 | 0 | 0 |
| Georgia | 0 | 0.002 | 0.038 | 0.083 | North Dakota | 0 | 0 | 0 | 0 |
| Hawaii | 0.422 | 0 | 0 | 0 | Ohio | 0 | 0.351 | 0 | 0 |
| Idaho | 0 | 0 | 0 | 0 | Oregon | 0 | 0 | 0 | 0 |
| Illinois | 0 | 0.076 | 0.062 | 0 | Pennsylvania | 0 | 0 | 0.311 | 0 |
| Indiana | 0 | 0 | 0 | 0 | Rhode Island | 0 | 0 | 0 | 0 |
| Kentucky | 0 | 0 | 0 | 0 | South Dakota | 0 | 0 | 0 | 0 |
| Louisiana | 0 | 0 | 0 | 0 | Tennessee | 0 | 0 | 0 | 0 |
| Maine | 0 | 0 | 0 | 0 | Utah | 0 | 0 | 0 | 0 |
| Massachusetts | 0 | 0 | 0 | 0 | Vermont | 0 | 0 | 0 | 0 |
| Michigan | 0 | 0 | 0 | 0 | Virginia | 0 | 0 | 0 | 0 |
| Minnesota | 0 | 0 | 0 | 0 | Washington | 0 | 0 | 0 | 0.169 |
| Montana | 0 | 0.032 | 0 | 0 | West Virginia | 0 | 0 | 0 | 0 |
| Nevada | 0 | 0 | 0 | 0 | Wyoming | 0 | 0 | 0 | 0 |

**Source**: 2010-2019 CDC Abortion Surveillance data. **Notes**: The base model uses each pre-period year outcome to generate the synthetic control unit. The alternative specifications use alternative sets of pre-period outcomes in combination with covariates in each pre-period year. 34 states are included in the synthetic control donor pool. Four states (CA, FL, MD, NH) and the District of Columbia are excluded due to missing data. Additionally, in this robustness test, six states (AZ, KS, MS, NE, OK, WI) are excluded as they have restrictive Title X funding policies, and five states (CO, IA, MO, SC, TX) are excluded as they had policy shocks that affect contraceptive access before or during the study period.

*B.3. Using an alternative approach to calculating synthetic control weights: Penalized synthetic control*

Lastly, we conducted penalized synthetic control analysis following Abadie & L’Hour (2020), to examine whether non-uniqueness is a potential issue in our model that influences the results and findings. When there is a large number of control units, non-unique, multiple solutions may exist. Although it rarely creates an issue when there is one treatment unit and when there’s a large set of covariates (which both are our case), we performed the penalized synthetic control analysis as a robustness check. Appendix Table B3-1. compares the synthetic control results in the main text with the penalized synthetic control result. For all the three specifications, the penalized synthetic control showed similar results as the models in our main text: slightly larger effects and smaller p-values in 2016 and in 2017, which attenuated in 2018, but all are not significant.

Appendix Table B3-1. Comparison of results: main model vs penalized synthetic model

|  | Treatment Effect, DE vs. synthetic DE | | P-value | |
| --- | --- | --- | --- | --- |
|  | Main model | Penalized synth | Main model | Penalized synth |
| **Base Model** |  |  |  |  |
| 2015 | -0.03 | 0.34 | 1.00 | 0.72 |
| 2016 | -1.97 | -1.65 | 0.57 | 0.43 |
| 2017 | -1.55 | -1.45 | 0.61 | 0.59 |
| 2018 | -0.29 | -0.36 | 1.00 | 0.96 |
| 2019 | 0.79 | 0.70 | 0.72 | 0.78 |
| **Specification 1** |  |  |  |  |
| 2015 | -0.65 | -0.30 | 1.00 | 0.83 |
| 2016 | -1.83 | -1.58 | 0.39 | 0.26 |
| 2017 | -1.85 | -1.54 | 0.39 | 0.30 |
| 2018 | -1.44 | -1.01 | 0.57 | 0.52 |
| 2019 | -0.77 | -0.22 | 1.00 | 0.89 |
| **Specification 2** |  |  |  |  |
| 2015 | -0.99 | -0.58 | 1.00 | 0.72 |
| 2016 | -2.13 | -1.84 | 0.26 | 0.22 |
| 2017 | -2.17 | -1.76 | 0.28 | 0.24 |
| 2018 | -1.71 | -1.09 | 0.50 | 0.37 |
| 2019 | -1.07 | -0.28 | 0.72 | 0.89 |
| **Specification 3** |  |  |  |  |
| 2015 | -0.33 | -0.05 | 1.00 | 1.00 |
| 2016 | -1.56 | -1.38 | 0.39 | 0.43 |
| 2017 | -1.49 | -1.32 | 0.37 | 0.48 |
| 2018 | -0.57 | -0.66 | 1.00 | 0.76 |
| 2019 | 0.30 | 0.19 | 1.00 | 0.96 |

*B.4. Handling missing Maryland and DC abortion counts by Delaware residents*

The CDC abortion series is missing data on 5 jurisdictions, including Maryland and DC. Maryland and DC are particularly problematic because they neighbor Delaware, and thus, it is more likely that patients might travel between Delaware and these states to obtain abortion services. Not only are they not available as control states, but the Delaware resident abortion count omits abortions occurring in those jurisdictions. While this remains a limitation of our analysis, we conducted a number of analyses to gauge how likely it is that their omission substantially biases are results.

First, we examined if including DC as a comparison state would alter our results. DC was missing for two years ((2012 and 2016). We linearly interpolated DC’s missing resident abortions and re-estimated our main results including DC in the donor pool of comparison states. Because Maryland was missing in all years, we could not similarly impute the counts in Maryland. Adding DC to the donor states did not change the pre-period matching weights (HI 0.422 and NY 0.578), and the result was not significant (p=0.74). Figures that are equivalent to Figures 2 and 3 are not shown as there was a very minimal difference.

| Appendix Table B4-1. Result summary with linear interpolating DC residents’ abortion counts and adding DC to the donor states (years 2012, 2016) | | | | |  |
| --- | --- | --- | --- | --- | --- |
|  |  | Average Post-Period Difference | P-Value | Goodness-of-fit |  |
|  |  |  |  |  |  |
| Base Model (main model) | | -0.61 | 0.71 | 0.96 |  |
| Base Model adding DC in the comparison state | | -0.65 | 0.74 | 0.96 |  |
| **Source**: 2010-2019 CDC Abortion Surveillance data. **Notes**: Abortion rate = number of abortions per 1,000 women ages 15-44. The base model uses each pre-period year outcome (2010-2014) to generate the synthetic control unit. Four states (CA, FL, MD, NH) are excluded from the synthetic control donor pool | | | | |  |

Second, the more important limitation is that abortions occurring in MD and DC to Delaware residents are not captured in the CDC data. Our main estimates simply ignore this issue which effectively assumes that 0 abortions to Delaware patients occur in DC and MD. Our main results are robust to departures from this assumption if the number of abortions to Delaware patients remains consistent from year to year.

However, it is plausible that the number of abortions to Delaware patients occurring in DC and MD did fluctuate over time. To gauge how likely it is that missing resident abortions to Delaware patients impacted our analysis, we re-estimated our models under 4 different scenarios, outlined below:

1. We imputed the number of Delaware abortions occurring in MD or DC using a ratio adjustment approach. Importantly, these imputed counts varied by year. The approach assumed that number of DE resident abortions occurring in those states was proportionate to the number of DE women who worked in those states and that the relationship between labor flows and abortion flows was consistent with New Jersey or Pennsylvania—two other states bordering Delaware. Specifically, we calculated the ratio of the share of employed DE women that worked in MD (or DC) to the share of employed DE women that worked in NJ (or PA) and then used that ratio to scale the number of DE resident abortions occurring in NJ (or PA) to obtain the imputed count of resident DE abortions occurring in MD and DC. The imputation used the following formula:

$$\frac{\% of employed DE women working in MD (or DC)}{\% of employed DE women working in PA (or NJ)}\times number of abortions occurred in PA \left( or NJ \right) by DE resident.$$

The DC counts were imputed only for 2012 and 2016, the years that they did not report to the CDC. Appendix Table B4-2 describes the imputed counts.

2. The approach described above is obviously sensitive to how well NJ or PA mimics MD or DC. To examine how sensitive our results are to that assumption, we examined various combinations of the PA-based and NJ-based imputations. We used the average of the two imputations (NJ or PA), twice of the average, half of the average, weighted average of 2/3*(PA-based imputation) + 1/3*( NJ-based imputation), and a weighted average of 1/3*(PA-based imputation) + 2/3*( NJ-based imputation).

3. For the District of Columbia, we re-ran our synthetic control models with a potential maximum number of DE resident abortions in DC, which we assumed 49, for the two missing years (2012, 2016). The reason for this assumption is that the numbers of DE resident abortions occurring in DC were less than 50 for all the non-missing years of DC.

4. We examined various combinations of different approaches to imputed DE resident abortions occurring in MD and DC and including DC in the donor pool of comparison states.

Appendix Table B4-3 presents synthetic control results using the range of sensitivity tests described above. Appendix Figure B4-1 presents synthetic control graphs for some of the methods that had a goodness-of-fit statistic over 0.8. None of the sensitivity tests indicated significant effects of DelCAN on abortions, which suggests the results described in the main paper are robust.

Appendix Table B4-2. Details on imputing Maryland and District of Columbia abortion counts by Delaware residents using information from Pennsylvania and New Jersey.

| **Panel A: Benchmark to Pennsylvania** | | | | | | |
| --- | --- | --- | --- | --- | --- | --- |
|  | Pennsylvania | | Maryland | | District of Columbia | |
| Year | % of DE women working in PA | # Abortions by DE residents | % of DE women working in MD | IMPUTED # Abortions by DE residents | % of DE women working in DC | IMPUTED # Abortions by DE residents |
| 2010 | 5.2 | 166 | 5.5 | 176 |  |  |
| 2011 | 6.2 | 271 | 3.9 | 170 |  |  |
| 2012 | 8.1 | 205 | 4.9 | 124 | 0.4 | 10 |
| 2013 | 7.8 | 304 | 2.8 | 109 |  |  |
| 2014 | 4.7 | 335 | 5.7 | 406 |  |  |
| 2015 | 6.6 | 405 | 3.8 | 233 |  |  |
| 2016 | 5.2 | 499 | 5.1 | 489 | 0 | 0 |
| 2017 | 5.8 | 612 | 5.2 | 549 |  |  |
| 2018 | 8.8 | 855 | 6.2 | 602 |  |  |
| 2019 | 6.8 | 815 | 3.5 | 419 |  |  |
| **Panel B: Benchmark to New Jersey** | | | | | | |
|  | New Jersey | | Maryland | | District of Columbia | |
| Year | % of DE women working in NJ | # Abortions by DE residents | % of DE women working in DE | IMPUTED # Abortions by DE residents | % of DE women working in DC | IMPUTED # Abortions by DE residents |
| 2010 | 0.5 | 60 | 5.5 | 660 |  |  |
| 2011 | 1 | 55 | 3.9 | 214 |  |  |
| 2012 | 0.6 | 72 | 4.9 | 588 | 0.4 | 48 |
| 2013 | 1.4 | 90 | 2.8 | 180 |  |  |
| 2014 | 0.7 | 104 | 5.7 | 847 |  |  |
| 2015 | 2.1 | 109 | 3.8 | 197 |  |  |
| 2016 | 0.3 | 113 | 5.1 | 1921 | 0 | 0 |
| 2017 | 1.5 | 128 | 5.2 | 444 |  |  |
| 2018 | 0.8 | 112 | 6.2 | 868 |  |  |
| 2019 | 1.4 | 111 | 3.5 | 278 |  |  |

Source: 2010-2019 CDC Abortion Surveillance data and 2010-2019 ACS.

| Appendix Table B4-3. Summary of Results with Various Ways of Imputing the number of abortions by Delaware residents occurred in Maryland and District of Columbia | | | |
| --- | --- | --- | --- |
|  | Average Post-Period Difference | P-Value | Goodness-of-fit |
| **A. Models presented in the main paper** |  |  |  |
| Base Model | -0.61 | 0.71 | 0.96 |
| Average of Alternative Specifications | -1.40 | 0.52 | 0.79 |
| **B. Imputing abortion counts conducted in MD by DE residents** |  |  |  |
| (1) MD counts proportional to PA | 0.82 | 0.78 | 0.94 |
| (2) MD counts proportional to NJ | 0.93 | 0.80 | 0.59 |
| (3) MD counts proportional to average of PA and NJ | 0.88 | 0.80 | 0.77 |
| (4) MD counts proportional to weighted average of PA and NJ (2/3:1/3) | 0.85 | 0.80 | 0.83 |
| (5) MD counts proportional to weighted average of PA and NJ (1/3:2/3) | 0.89 | 0.80 | 0.71 |
| (6) MD counts proportional to twice of average of PA and NJ | 2.71 | 0.80 | 0.48 |
| (7) MD counts proportional to twice of PA | 2.05 | 0.72 | 0.85 |
| (8) MD counts proportional to twice of NJ | 3.37 | 0.83 | 0.26 |
| (9) MD counts proportional to half of average of PA and NJ | 0.31 | 0.80 | 0.90 |
| (10) MD counts proportional to half of PA | 0.26 | 0.76 | 0.97 |
| (11) MD counts proportional to half of NJ | 0.33 | 0.80 | 0.81 |
| **C. Imputing abortion counts conducted in DC by DE residents for DC non-participation years (2012, 2016)** |  |  |  |
| (1) DC counts proportional to PA | -0.61 | 0.74 | 0.96 |
| (2) DC counts proportional to NJ | -0.64 | 0.76 | 0.94 |
| (3) DC counts as 49 abortions (potential max) | -0.59 | 0.80 | 0.94 |
| **D. Combinations of above** |  |  |  |
| 1. MD as B-1 & DC as C-1 | 0.82 | 0.78 | 0.94 |
| 1. MD as B-1 & DC as C-2 | 0.82 | 0.80 | 0.92 |
| 1. MD as B-1 & DC as C-3 | 0.93 | 0.80 | 0.92 |
| 1. MD as B-10 & DC as C-1 | 0.25 | 0.78 | 0.96 |
| 1. MD as B-10 & DC as C-2 | 0.21 | 0.80 | 0.95 |
| 1. MD as B-10 & DC as C-3 | 0.27 | 0.80 | 0.95 |
| 1. MD as B-1 & DC as C-1 & interpolated DC in the donor pool | 0.82 | 0.79 | 0.94 |
| 1. MD as B-10 & DC as C-1& interpolated DC in the donor pool | 0.25 | 0.79 | 0.96 |
| Source: 2010-2019 CDC Abortion Surveillance data. Abortion rate = the number of abortions per 1,000 women aged 15-44. The models use each pre-period year outcome to generate the synthetic control unit. P-values come from permutations tests. Goodness-of-fit is a measure of the quality of the match and is defined as pre-treatment normalized mean squared error. Four states (CA, FL, MD, NH) are excluded due to missing data. The District of Columbia is also excluded, except for models D-7 and D-8. | | | |

| Appendix Figure B4-1. Synthetic Control Results with Imputations on MD and DC | |
| --- | --- |
| Panel a. Base model (main model) | Panel b. Model B-1 |
| 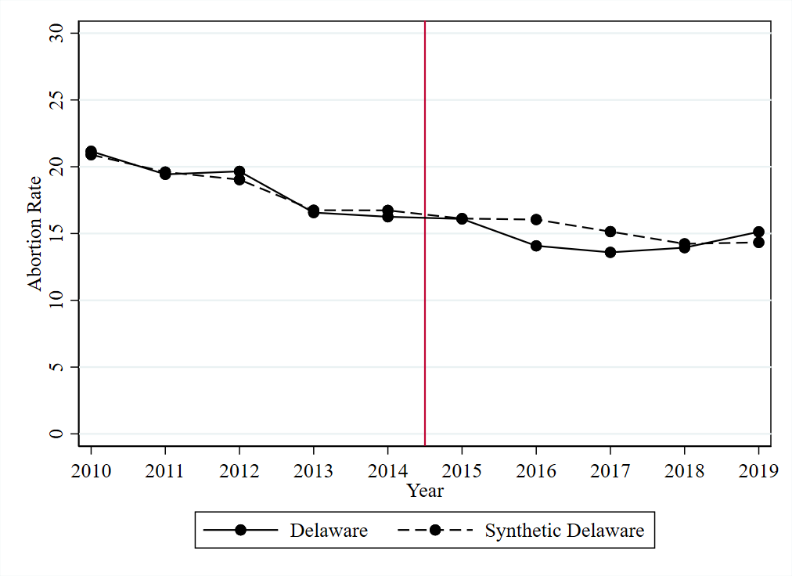 | **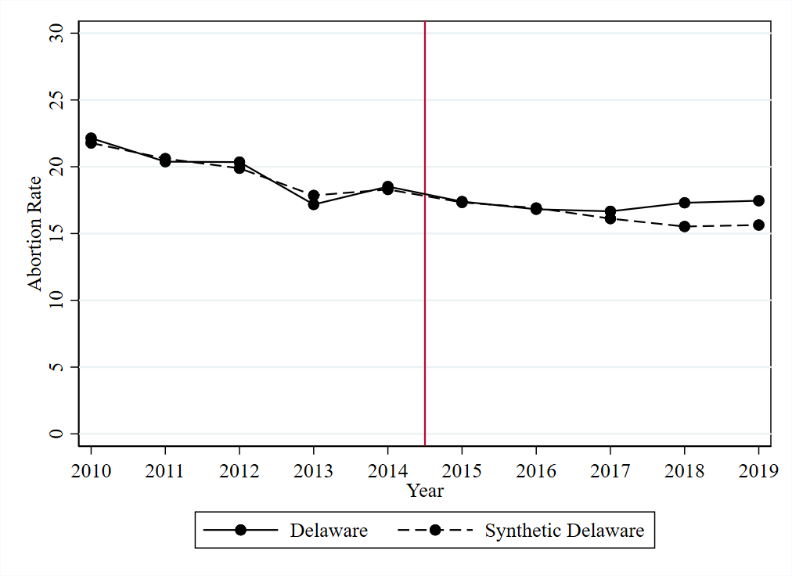** |
| Panel c. Model B-9  **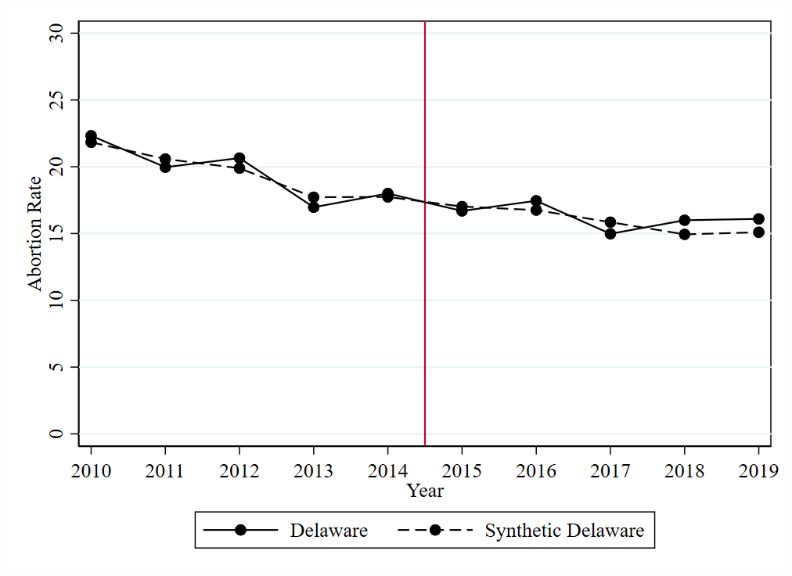** | Panel d. Model B-10  **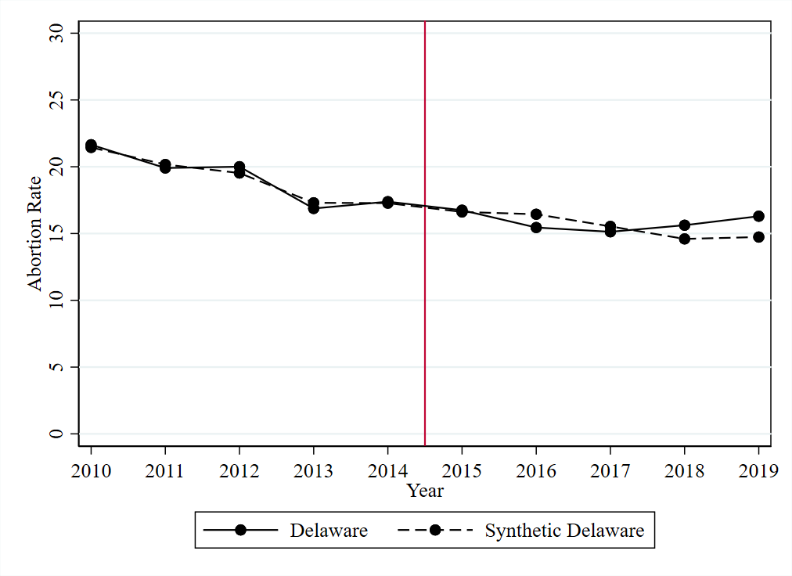** |
| Panel e. Model D-4  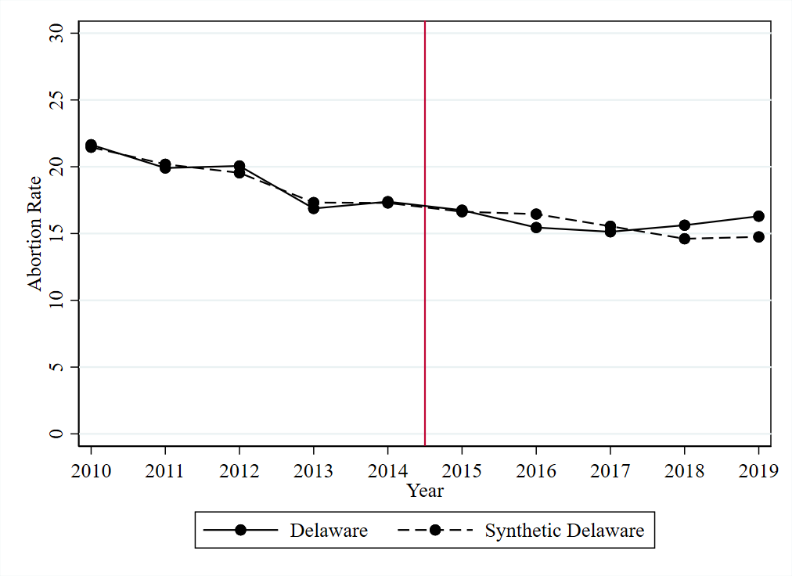 | Panel f. Model D-7  **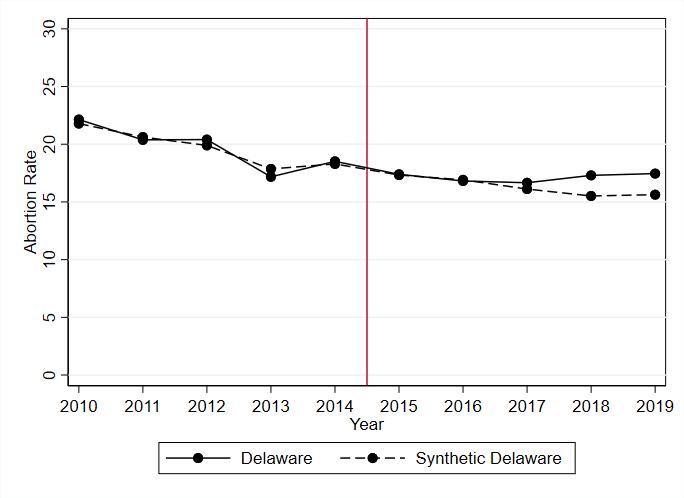** |
| Source: 2010-2019 CDC Abortion Surveillance data. Abortion rate = the number of abortions per 1,000 women aged 15-44. The models use each pre-period year outcome to generate the synthetic control unit. P-values come from permutations tests. Goodness-of-fit is a measure of the quality of the match and is defined as pre-treatment normalized mean squared error. Four states (CA, FL, MD, NH) are excluded due to missing data. The District of Columbia is also excluded, except for models D-7 and D-8. | |

**Appendix C. Abortion Data from the CDC and Guttmacher Institute**

The CDC data could be downwardly biased for a number of reasons (Kortsmit et al., 2020, 2021). The voluntary nature of the CDC Abortion Surveillance System leads to completely missing data for some states for all or some years such as Maryland, DC, California, and New Hampshire and to underreporting in participating states when patients travel between states to obtain services. Even when states do report, the states do not always require that all providers report. For example, New Jersey only collect reports from hospitals and licensed ambulatory facilities (Kortsmit et al., 2021).

An alternative to the CDC counts are counts produced by the Guttmacher Institute based largely on their Abortion Provider Census (APC) (Maddow-Zimet & Kost, 2021). Counts are produced for all states, and the APC contacts all healthcare facilities known or suspected to provide abortion care in the US (Jones et al., 2022).

Comparing 2019 CDC data to 2019 Guttmacher data, the state-level CDC counts were 88% of the Guttmacher counts on average. However, there is heterogeneity across the states. Counts in 12 states were 47-82% of alternative estimates produced by the Guttmacher Institute, while the rest of the states had 88% or higher of the Guttmacher estimates (Jones et al., 2022; Kortsmit et al., 2021).

While Guttmacher’s abortion data clearly have advantages, they also have important limitations. They likely have both response and non-response errors, and we are unaware of any empirical evidence that the error in the CDC estimates exceeds the error in the Guttmacher estimates. While the CDC system is voluntary for the states, states can mandate that providers report patient volumes, as Delaware has done since 1997 (Delaware Health Statistics Center, 2021). Enforcement can be based on state licensure lists of mandated reporters. Guttmacher must build its abortion provider list independently. It does so by starting with the known universe from the previous Census wave and updating it based on web searches, media reports, and professional association membership lists (Jones et al., 2022). While their approach is surely comprehensive, it seems plausible that it misses providers, and we are unaware of any study that validates it.

Once their list is compiled, they have no ability to mandate participation in the APC and only a fraction of providers participate. In the most recent APC, Guttmacher obtained responses from 52% of providers. Counts from missing providers are obtained from state health departments (17% of providers) or by estimating the count (31%) based on “prior surveys, key informants, media stories, on-line reviews, and other tools” (Jones et al., 2022).

Perhaps the most important limitation of the Guttmacher estimates for our purpose is that the data is not collected for every year (Jones et al., 2022; Maddow-Zimet & Kost, 2021). The APC is conducted for two out of every three years; for example, data were collected for 2013 and 2014, and 2015 was skipped, then data for 2016 and 2017 were collected, and so on. Given that our study design leverages year-to-year variation, this is an important concern. Appendix Figure C1 compares the abortion rates from the CDC Abortion Surveillance System and Guttmacher Institute. Three years (2012, 2015, 2018) out of ten years for our main analysis time frame are missing from the Guttmacher data.

Appendix Figure C1 illustrates two other important features of the Guttmacher data. First, the difference between the CDC and Guttmacher is substantial in Delaware during the pre-period. However, this gap diminishes over time and is small and consistent starting in 2013. In contrast, the difference between the CDC and the Guttmacher is small in the comparison states in all years. Indeed, the gap in Delaware appears to converge to the average state gap over time. Second, the Guttmacher data suggests a large decline in abortion rates in Delaware between 2007 and 2013. While there is some decline apparent in the CDC data, it is much more moderate. We are unaware of any policy, practice, or social factor that might explain the large declines observed in the Guttmacher series. Combined with the convergence in the gap in Delaware between the CDC and Guttmacher data, it is plausible that the pattern in Appendix Figure C1 are explained by changes in measurement error in the Guttmacher data, perhaps due to changes in coverage error or non-response. Given the small nature of the state, a small number of non-covered or non-responding providers could have a large effect on the count. This of course is a conjecture, and it could be that measurement error in the CDC data is changing over time. However, based on conversations with state officials in Delaware, we are unaware of any systematic change in reporting that could be responsible for such change.

| Appendix Figure C1. Abortion Rates from the CDC Abortion Surveillance vs. the Guttmacher Institute | |
| --- | --- |
| Panel a. Delaware | Panel b. Average of control states |
| **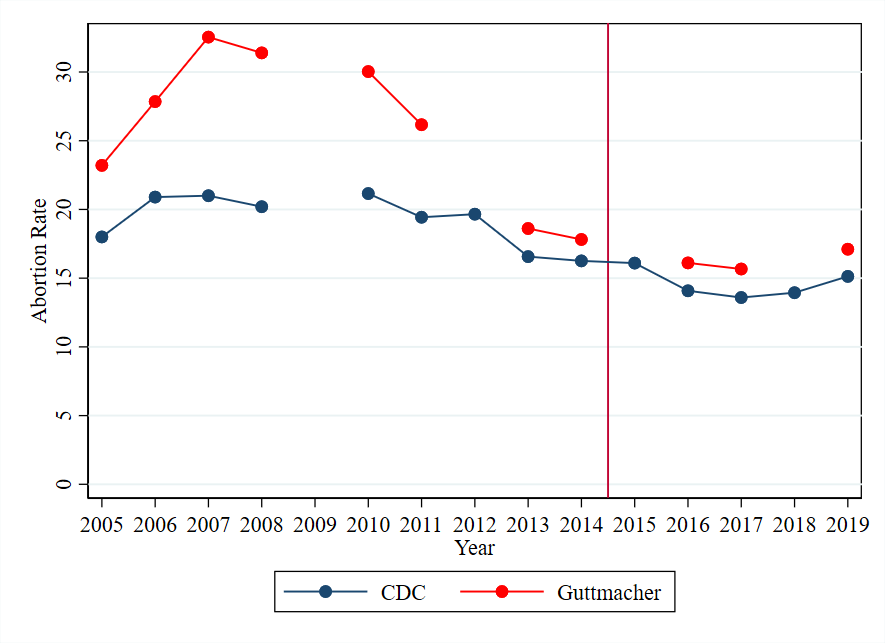** | **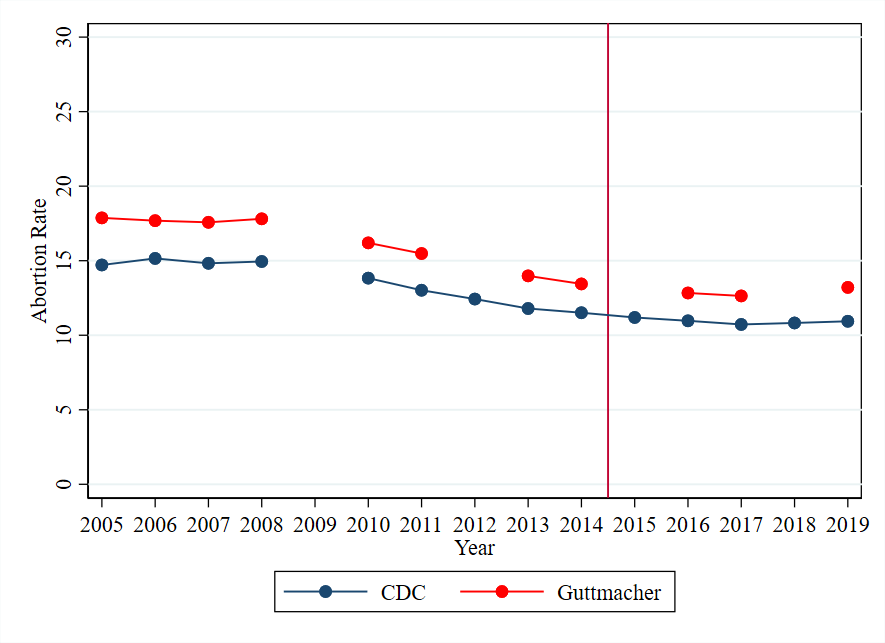** |

**Source**: 2005-2019 CDC Abortion Surveillance data & 2005-2019 Guttmacher abortion data. **Notes**: Abortion rate = number of abortions per 1,000 women ages 15-44. The control states are excluding Delaware, and six states (CA, FL, KY, LA, MD, NH) and the District of Columbia due to missing data.

Despite our reservations about Guttmacher’s estimates for our specific study, it is reasonable to replicate our synthetic control results using the Guttmacher data. Appendix Figure C2 shows the synthetic control results using Guttmacher Institute’s data 2005-2019, which is analogous to the analysis shown in Appendix section B.3 using the same control states as the CDC model. Appendix Figure C3 shows the results using all states for the control set, utilizing the Guttmacher data’s strength.

It is clear from Appendix Figures C2 and C3 that we are unable to obtain a suitable match for Delaware. The goodness-of-fit from the models were 0.63 (Appendix Figure C2; CDC states as control) and 0.64 (Appendix Figure C3; all states as control), which is substantially lower compared to the models using CDC data (Appendix B.3. model: 0.8, model in the main paper: 0.96). We attempted to improve the model fit starting from different years (2010) and using linearly interpolated data for the missing years, but the poor fit was consistent in all specifications.

We suspect this poor fit is caused by the fact that Guttmacher series suggests that Delaware experienced extreme changes to abortion rates in the pre-period and no combination of donor pool states can adequately capture that change. The much more moderate changes in Delaware in the CDC series can be captured by a combination of donor states. The poor match in the Guttmacher data suggests substantial caution in drawing conclusions from the synthetic control comparisons. However, similar to results based on the CDC data, we fail to find a significant effect. For the model of Appendix Figure C2 (CDC states as control), the average post-period difference is 2.48 fewer abortions per 1,000 women, and the p-value is 0.86. For the model of Appendix Figure C3 (all states as control), the average post-period difference is 2.46 fewer abortions per 1,000 women, and the p-value is 0.94.

| Appendix Figure C2. Synthetic Control Results Using Guttmacher Data (2005-2019), Delaware vs. Synthetic Delaware (Control group with CDC states) | |
| --- | --- |
| **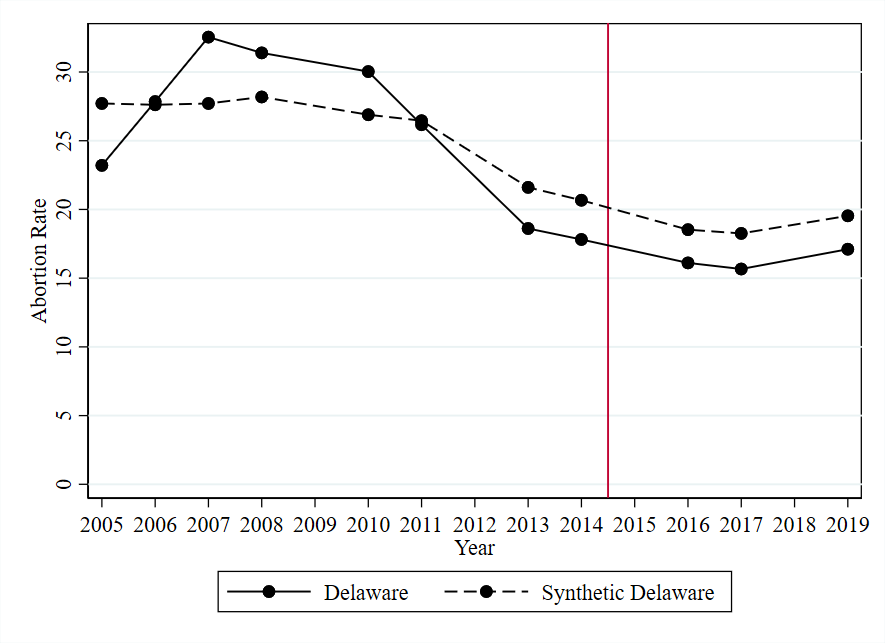** |  |
| **Source**: 2005-2019 Guttmacher Institute’s abortion data.  **Notes**: Abortion rate = number of abortions per 1,000 women ages 15-44. The model used base model with each pre-period year outcome (2005-2008 and 2010-2014) to generate the synthetic control unit. Six states (CA, FL, KY, LA, MD, NH) and the District of Columbia are excluded due to missing data. The set of control states is the same as the model using CDC data (analogous to Appendix Figure B3). Two states (HI and NY) contribute positive weight to synthetic Delaware. |  |

| Appendix Figure C3. Synthetic Control Results Using Guttmacher Data (2005-2019), Delaware vs. Synthetic Delaware (Control group with all states) | |
| --- | --- |
| **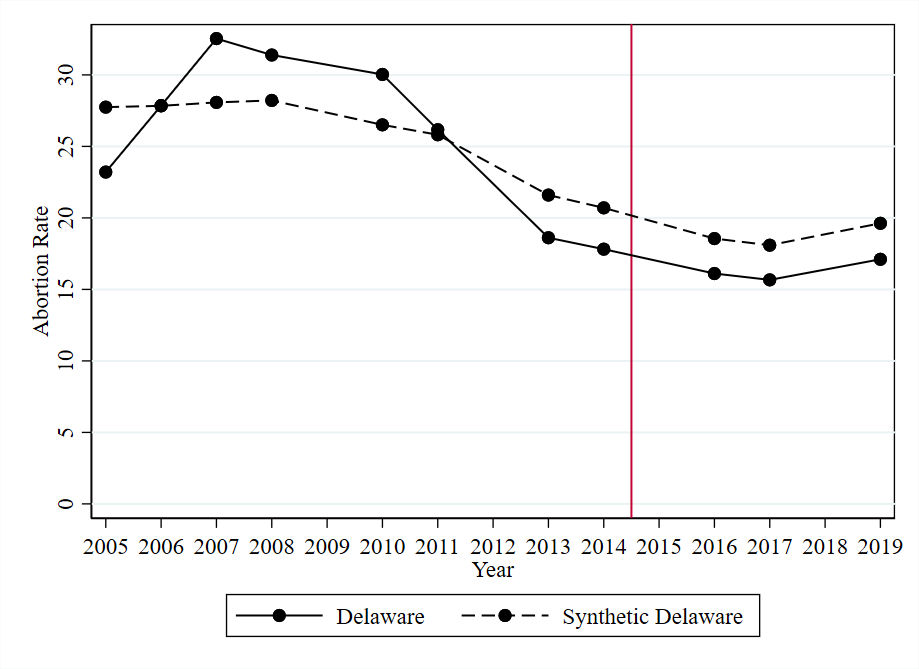** |  |
| **Source**: 2005-2019 Guttmacher Institute’s abortion data.  **Notes**: Abortion rate = number of abortions per 1,000 women ages 15-44. The model used base model with each pre-period year outcome (2005-2008 and 2010-2014) to generate the synthetic control unit. All states but Delaware were included in the control set. Three states (CA, HI, NY) contribute positive weight to synthetic Delaware. |  |

**Reference**

Abadie, A., Diamond, A., & Hainmueller, J. (2010). Synthetic Control Methods for Comparative Case Studies: Estimating the Effect of California’s Tobacco Control Program. *Journal of the American Statistical Association*, *105*(490), 493–505. https://doi.org/10.1198/jasa.2009.ap08746

Abadie, A., & L’Hour, J. (2020). A Penalized Synthetic Control Estimator for Disaggregated Data. *Working Paper*, 1–35.

Colorado Department of Public Health and Environment. (2017). *Colorado’s Success With Long-Acting Reversible Contraception*. www.colorado.gov/cdphe/cfpi-report%0Ahttps://www.colorado.gov/pacific/sites/default/files/PSD_TitleX3_CFPI-Report.pdf

Delaware Health Statistics Center. (2019). *Delaware Vital Statistics Annual Report 2018*. https://www.dhss.delaware.gov/dhss/dph/hp/files/ar2018_net.pdf

Delaware Health Statistics Center. (2021). *Delaware Vital Statistics Annual Report 2019: Technical Notes*. https://doi.org/10.1300/J184v06n04_07

Guttmacher Institute. (2022, May 1). *State Family Planning Funding Restrictions*. Guttmacher Institute. https://www.guttmacher.org/state-policy/explore/state-family-planning-funding-restrictions

Jones, R. K., Kirstein, M., & Philbin, J. (2022). Abortion incidence and service availability in the United States, 2020. *Perspectives on Sexual and Reproductive Health*, *54*(4), 128–141. https://doi.org/10.1363/psrh.12215

Kortsmit, K., Jatlaoui, T. C., Mandel, M. G., Reeves, J. A., Oduyebo, T., Peterson, E., & Whiteman, M. K. (2020). Abortion surveillance - United States, 2018. *MMWR Surveillance Summaries*, *69*(7), 1–29. https://doi.org/10.15585/mmwr.ss6907a1

Kortsmit, K., Mandel, M. G., Reeves, J. A., Clark, E., Pagano, H. P., Nguyen, A., Petersen, E. E., & Whiteman, M. K. (2021). Abortion Surveillance — United States, 2019. *MMWR. Surveillance Summaries*, *70*(9), 1–29. https://doi.org/10.15585/mmwr.ss7009a1

Maddow-Zimet, I., & Kost, K. (2021). Pregnancies, Births and Abortions in the United States, 1973–2017: National and State Trends by Age—Methodology Appendix. In *Guttmacher Institute*. https://doi.org/10.4337/9780857938336.00019

McNicholas, C., Tessa, M., Secura, G., & Peipert, J. F. (2014). The Contraceptive CHOICE Project Round Up: what we did and what we learned. *Clinical Obstetrics and Gynecology*, *57*(4), 635–643. https://doi.org/10.1097/GRF.0000000000000070.

Philliber Research Associates. (2010). *Evaluating the Iowa Initiative to Reduce Unintended Pregnancies*.

Sundstrom, B., Billings, D., Smith, E., Ferrara, M., Albert, B., & Suellentrop, K. (2019). Evaluating the Whoops Proof S.C. Campaign: A Pair-Matched Group Pretest–Posttest Quasi-experimental Study. *Maternal and Child Health Journal*, *23*(8), 1036–1047. https://doi.org/10.1007/s10995-018-02729-6

Sundstrom, B., Billings, D., & Zenger, K. E. (2016). Keep Calm and LARC On: A theory-based long-acting reversible contraception (LARC) access campaign. *Journal of Communication in Healthcare*, *9*(1), 49–59. https://doi.org/10.1080/17538068.2016.1143165

White, K., Hopkins, K., Aiken, A. R. A., Stevenson, A., Hubert, C., Grossman, D., & Potter, J. E. (2015). The impact of reproductive health legislation on family planning clinic services in Texas. *American Journal of Public Health*, *105*(5), 851–858. https://doi.org/10.2105/AJPH.2014.302515
